# Spider minor ampullate silk protein nanoparticles: an effective protein delivery system capable of enhancing systemic immune responses

**DOI:** 10.1002/mco2.573

**Published:** 2024-06-15

**Authors:** Hairui Yu, Gefei Chen, Linchao Li, Guoqiang Wei, Yanan Li, Sidong Xiong, Xingmei Qi

**Affiliations:** ^1^ The Jiangsu Key Laboratory of Infection and Immunity Institutes of Biology and Medical Sciences Soochow University Suzhou China; ^2^ Department of Biosciences and Nutrition Karolinska Institutet Huddinge Sweden; ^3^ Department of Neurosurgery Changhai Hospital Naval Medical University Shanghai China

**Keywords:** immune response, nanoparticles, protein controlled release, spidroin

## Abstract

Spider silk proteins (spidroins) are particularly attractive due to their excellent biocompatibility. Spider can produce up to seven different types of spidroins, each with unique properties and functions. Spider minor ampullate silk protein (MiSp) might be particularly interesting for biomedical applications, as the constituent silk is mechanically strong and does not super‐contract in water, attributed to its amino acid composition. In this study, we evaluate the potential of recombinant nanoparticles derived from *Araneus ventricosus* MiSp as a protein delivery carrier. The MiSp‐based nanoparticles were able to serve as an effective delivery system, achieving nearly 100% efficiency in loading the model protein lysozyme, and displayed a sustained release profile at physiological pH. These nanoparticles could significantly improve the delivery efficacy of the model proteins through different administration routes. Furthermore, nanoparticles loaded with model protein antigen lysozyme after subcutaneous or intramuscular administration could enhance antigen‐specific immune responses in mouse models, through a mechanism involving antigen‐depot effects at the injection site, long‐term antigen persistence, and efficient uptake by dendritic cells as well as internalization by lymph nodes. These findings highlight the transnational potential of MiSp‐based nanoparticle system for protein drug and vaccine delivery.

## Graphical abstract

This study displayed the potential use of spider MiSp‐derived NM‐NPs as a nanocarrier for protein antigens. The mechanisms underlying how NM‐NPs act as a vaccine carrier to promote antigen‐specific immune responses are multifaceted. (1) High loading efficiency of antigen, (2) the nanoparticles establish an antigen depot at the injection site, (3) efficient uptake by APCs in the draining lymph node, (4) promote DC maturation in the draining lymph nodes and promoting cross‐presentation, and (5) significant increases in specific antibodies levels and cellular immune responses.

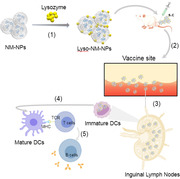

## 1 INTRODUCTION

Spider silk is known for its exceptional strength, toughness, and elasticity, as well as its biodegradability and biocompatibility, which make it an intriguing material for a range of applications in medicine, textiles, and industry.^1^ Spider silk is built by spider silk proteins (spidroins), including major ampullate silk protein (MaSp), minor ampullate silk protein (MiSp), and aciniform silk protein responsible for the production of their respective silk types.^2^ Although different spidroins share a common structure, including a large repetitive central region flanked by nonrepetitive globular N‐ and C‐terminal domains (NT and CT), the amino acid sequences of the repetitive central region differ significantly among different spidroins, which determines the unique properties of each silk type.^3^, ^4^ Spidroins have garnered significant attention due to their desirable characteristics, including biocompatibility, biodegradability, and the ability to induce only a mild immune response.^5^, ^6^, ^7^, ^8^, ^9^, ^10^, ^11^, ^12^ A deeper understanding of the structure and function of spidroins has led to the development of silk‐based materials with novel functionalities, beyond fiber formation, such as films, hydrogels, sponges, scaffolds, and nanoparticles (NPs).^13^

Silk‐derived materials, with their versatility and biochemical properties, have the potential to be suitable for tissue engineering and as carriers of protein drugs, nucleic acids, and peptide vaccines.^11^, ^14^ In particular, NPs derived from spidroins can be prepared without solvents and sterilized with steam.^10^ Additionally, their size, stability, and drug loading/release kinetics are able to be easily tailored by adjusting the particle formation process and posttreatment of the material.^15^, ^16^ So far, NP formation of spidroins has primarily centered on eADF4(C16), an engineered MaSp2 variant composed of sixteen repetitive C‐modules (C‐module amino acid sequence: GSSAAAAAAAASGPGGYGPENQGPSGPGGYGPGGPG). When exposed to high concentrations of potassium phosphate, recombinant eADF4(C16) can self‐assemble into chemically stable microspheres.^15^ The resulting particle size ranges from a few hundred nanometers to approximately 2 µm,^16^ and small molecules and macromolecular protein have been incorporate into eADF4(C16) particles.^17^ Using lysozyme as a model protein, a high loading efficiency was achieved, and the release of lysozyme was dependent on the ionic strength and pH.^10^ The eADF4(C16) particles were also utilized as carriers, incorporating a short peptide antigen (amino acid sequence: SIINFEKL). The prepared hybrid protein particles were taken up by dendritic cells (DCs), leading to successful activation of cytotoxic T‐cells, and no signs of immunotoxicity or unspecific immunostimulatory activity were observed.^12^ Furthermore, recombinant customized spidroins MS1 and MS2 derived from the repetitive regions of MaSp1 and MaSp2 formed spheres, respectively. Interestingly, the MS2 carried a negative charge, in contrast to the positive charge of MS1 spheres. These differences affected the suitability of MS1 and MS2 spheres for various drug applications, highlighting the importance of selecting the appropriate spidroin for a given purpose.^18^ Based on these observations, it is evident that NPs formed by various spidroins possess distinct properties, each with unique properties suitable for diverse biological applications. Attributed to the amino acid composition that mainly consists of Gly and Ala, spider minor ampullate silk may be particularly interesting for biomedical applications, since it is mechanically strong, does not super‐contract in water, and fibrillizes through a non‐nucleation dependent pathway.^19^, ^20^

Compared with inactivated or attenuated whole‐cell vaccines, subunit vaccines containing protein or polypeptide fragments may offer greater safety as a new generation of vaccine candidates.^21^ However, protein vaccines alone are often poorly immunogenic due to factors such as rapid degradation by proteases and promoting tolerance. As a potential alternative, antigens can be protected by particulate carriers. For instance, particulate‐based carriers can deliver antigens to antigen‐presenting cells (APCs) and regulate the antigen presentation pathway or act as immune potentiators, enhancing subsequent antigen‐specific immune responses.^22^, ^23^

Considering the intrinsical advantages of MiSp, in this study, we focus on a customized MiSp version and assess the formation of NPs, and their biocompatibility and the potential applications as protein delivery carriers. This customized spidroin encompasses a segment of the low complexity region (M) and the nonrepetitive NT domain that has not yet introduced in so far studied NPs. Additionally, the potential of MiSp‐based NPs (NM‐NPs) as a delivery system for subunit protein vaccine was also explored in mouse models.

## 2 RESULTS

### 2.1 MiSp‐based NP preparation and characterization

**FIGURE 1:**
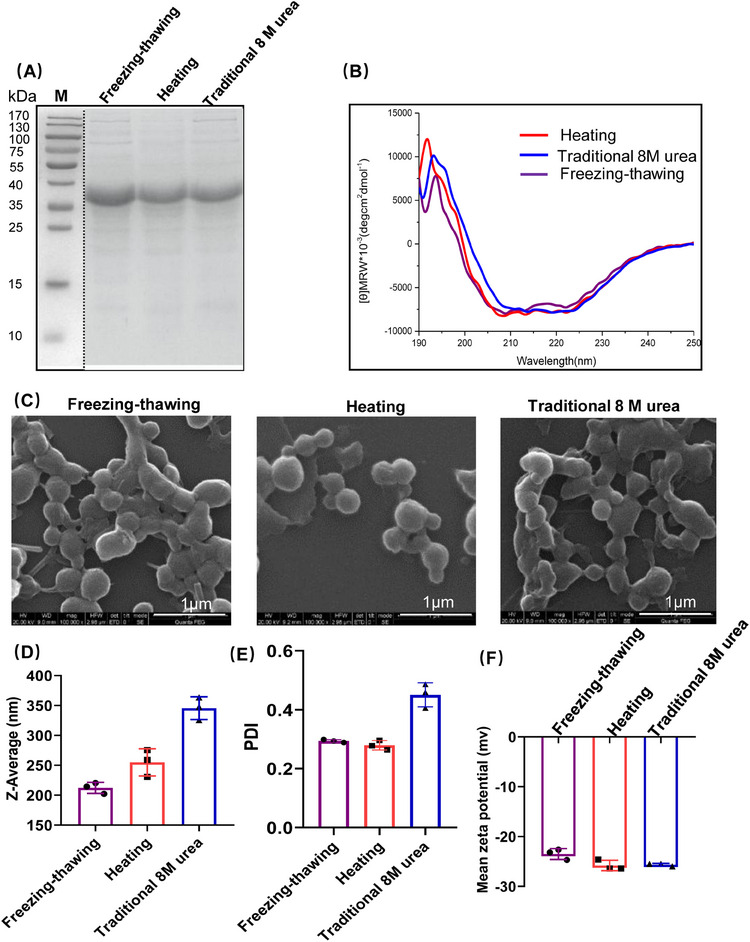
Preparation and characterization of NM‐NPs. (A) NM IBs solubilization with different methods, that is, freezing–thawing with 2 M urea, one‐step heating with 4 M urea, and traditional 8 M urea‐denaturation methods, and analyzed by SDS‐PAGE. (B) CD spectra of the NM proteins obtained by different solubilization methods. (C) Characterization of NM‐NPs with SEM, the scale bar (1 µm) is shown in each image. (D) Diameters of NM‐NPs derived from recombinant proteins obtained by the three different methods (*n* = 3). (E and F) Polydispersity index (PDI) and zeta potential of NM‐NPs obtained by the three different methods analyzed by DLS (*n* = 3). Data represent mean ± SD of three independent experiments.

The process of forming NPs by salting out using potassium phosphate is widely regarded as the most biocompatible approach for producing silk protein NPs. Nonetheless, numerous factors, such as protein concentration, phosphate concentration, pH of the potassium phosphate solution, protein purification method, and amino acid sequence, can impact the properties of the resultant protein spheres.^15^, ^16^, ^24^, ^25^ With the goal of identifying appropriate NPs for utilization as potential delivery carriers, the influence of inclusion bodies (IBs) solubilization approaches on NP properties were subsequently explored. SDS‐PAGE (sodium dodecyl sulfate polyacrylamide gel electrophoresis) analysis indicated that all three methods generated recombinant NM proteins with good purity and high solubilization efficiency (Figure 1A), resulting in a final yield of approximately 50 mg per liter of LB medium. Circular dichroism (CD) measurements showed that soluble NM proteins from the above approaches shared similar secondary structures (Figure 1B), dominated by α‐helix that is probably due to the α‐helix bundle conformation of the NT domain.^26^ Revealed by scanning electron microscopy (SEM), despite being prepared using different approaches, the recombinant NM proteins were all able to form NPs (NM‐NPs), which were spherical in shape and exhibited a smooth surface (Figure 1C). However, the mean diameters of the NPs were diverse, 211.2 ± 9.1, 299.8 ± 8.1, and 355.6 ± 7.9 nm for freezing–thawing, heating, and traditional methods, respectively (Figure 1D and Table S1). These diameters are smaller than previously reported spidroin (eADF4)‐derived NP size,^15^, ^16^ which might be due to the different amino acid composition, while in this study the NT domain is included and the M mainly consists of Gly and Ala. Further, the freezing–thawing and heating methods generated more homogeneous NPs than the traditional 8 M urea method, indicated by the polydispersity index (Figure 1E and Table S1). Despite of these, all three types of NPs exhibited similar negative zeta potentials as measured by dynamic light scattering (DLS) (Figure 1F and Table S1). This observation suggests that the negatively charged N‐terminal domain (with a pI of 4.1) might be situated on the peripheral side, while the repetitive region M likely forms the core. Based on these observations, it is evident that the various IBs solubilization approaches had discernible effects on the size and homogeneity of the spheres. Taking into account the protein preparation process, sphere characteristics, and the possibility of urea modification, the freezing–thawing method was chosen as the standard method to produce NM proteins for NP preparation for subsequent applications.

### 2.2 Model protein loading, activity, and releasing behavior of NM‐NPs

**FIGURE 2:**
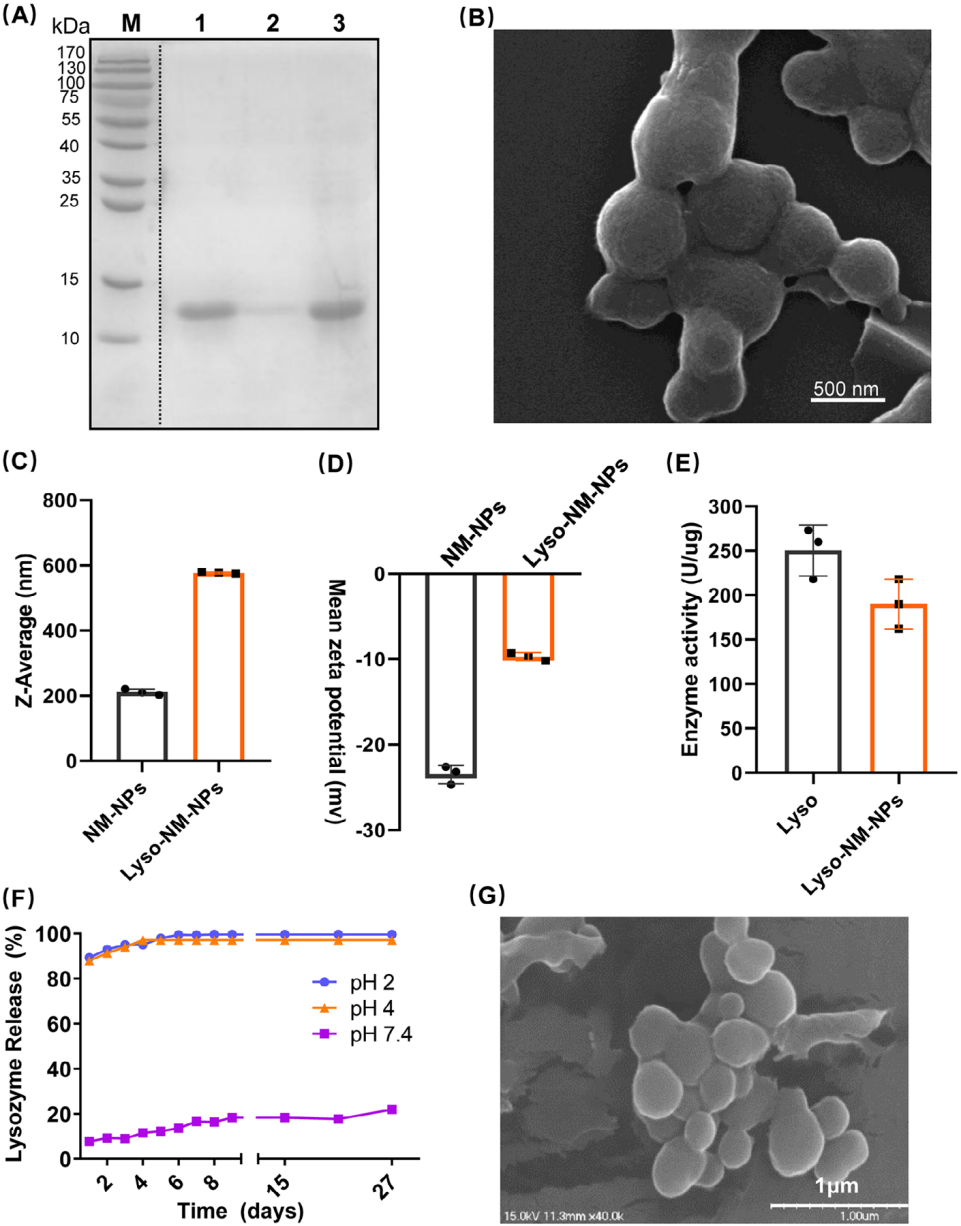
Loading, releasing and activity of lysozyme on NM‐NPs. (A) SDS‐PAGE analysis of the lysozyme loading on NM‐NPs. NM‐NPs were mixed with lysozyme for 2 h with a ratio of 1:10 (w/w). Lane 1, the total lysozyme protein before loading to NM‐NPs. Lane 2, the unbound protein in the supernatant. Lane 3, the lysozyme released from NM‐NPs. (B) SEM images of NM‐NPs after loaded with lysozyme. (C and D) Diameters and zeta potential NM‐NPs loaded with lysozyme (Lyso‐NM‐NPs) (*n* = 3). (E) Lysozyme activity measured by turbidimetric method (*n* = 3). (F) In vitro release profiles of lysozyme from Lyso‐NM‐NPs at different pHs. (G) SEM image of NM‐NPs after treated in SGFs for 24 h. Data represent mean ± SD of three independent experiments.

As NM‐NPs are negatively charged at a neutral pH, they could probably form complexes with positively charged molecules via electrostatic interactions. As such, the lysozyme with an isoelectric point of 11.35 and a molecular weight of 14.3 kDa was selected as a model protein. Lysozyme could be efficiently loaded on NM‐NPs with loading efficiency nearly 100% and loading at 10% (w/w‐ratios) of lysozyme:NM‐NPs of 1:10 (Table S2). The SDS‐PAGE analysis further confirmed the successful loading of lysozyme on NM‐NPs (Lyso‐NM‐NPs), as indicated by the tiny amount of free lysozyme left in the solution (Figure 2A). SEM observations showed that the Lyso‐NM‐NPs were uniform and spherical (Figure 2B), but with an increased average size of 510 ± 10.3 nm (Figure 2C) and a decreased negative zeta potential of −9.64 ± 0.5 mV compared with NM‐NPs (Figure 2D). The increased size and decreased zeta potential could be probably explained by the lysozyme surface loading on the particles and charge compensation by the opposite charge of lysozyme.

We also studied whether the Lyso‐NM‐NPs affected the lysozyme activity due to the protein encapsulation. The activity of lysozyme in NM‐NPs was evaluated using a common turbidimetric method with Lysozyme (LZM) Assay kit (S1.6, Supporting Information). As shown in Figure 2E, the activity of lysozyme in NM‐NPs was not significantly different from that of free lysozyme, with an activity of about 25,000 and 19,000 units/mg, respectively.

To monitor the release kinetics of loaded lysozyme from Lyso‐NM‐NPs, we tested the in vitro releasing efficiency at different pH levels (Figure 2F). Interestingly, at both pH 2.0 and 4.0, almost 90% of loaded lysozyme proteins were released immediately during the incubation from Lyso‐NM‐NPs, which is likely due to that the electrostatic interactions were broken up by low pHs. Conversely, Lyso‐NM‐NPs exhibited sustained lysozyme‐release behavior at pH 7.4, with around 22% of preloaded lysozyme released over a period of 27 days, suggesting NM‐NPs support sustained release of loaded protein in vitro, which is an important factor for reduced dosage and frequency from the immunization point of view. These results indicate positively changed macromolecular proteins could be loaded on NM‐NPs efficiently with minor activity impact, which further present a pH‐sensitive releasing profile and supports a sustained release behavior under neutral pH.

Finally, the degradation behavior of NM‐NPs was characterized by immersion tests in simulated gastric fluids (SGFs) (S1.7, Supporting Information). The morphology of the NM‐NPs was studied with SEM after a static immersion test in a water bath incubator at 37°C for 24 h. As shown in Figure 2G, it is clear that the presence of the organic molecules in the SGFs did not affect the morphology of the particles. The results indicated that the NM‐NPs was stable in SGFs.

### 2.3 Biodistribution and delivery efficiency of NM‐NPs in vivo

To comprehensively assess the possibility of NM‐NPs as a viable and effective protein carrier, the in vivo biodistribution and the delivery effects of protein drugs were evaluated when administrated through different pathways, including parenteral (subcutaneous [s.c.], intramuscular [i.m.], and intravenous [i.v.] injections) and nonparenteral (per‐oral) administration routes. To track the NPs or protein in vivo, different formulations NM‐NPs (Cy5.5 labeled), Lyso‐NM‐NPs (Cy5.5‐labeled Lyso), or free lysozyme (Lyso, Cy5.5 labeled) were administrated in Balb/c mice (Table S3), while mice administrated with PBS were as control and analyzed by the noninvasive imaging modality that allows the visualization of biological processes in living organisms using fluorescent Cy5.5, a near‐infrared fluorescent dye clinically used in medical diagnostics (Table S4 and Figure S1).

#### 2.3.1 Delivery effect for different administrations

**FIGURE 3:**
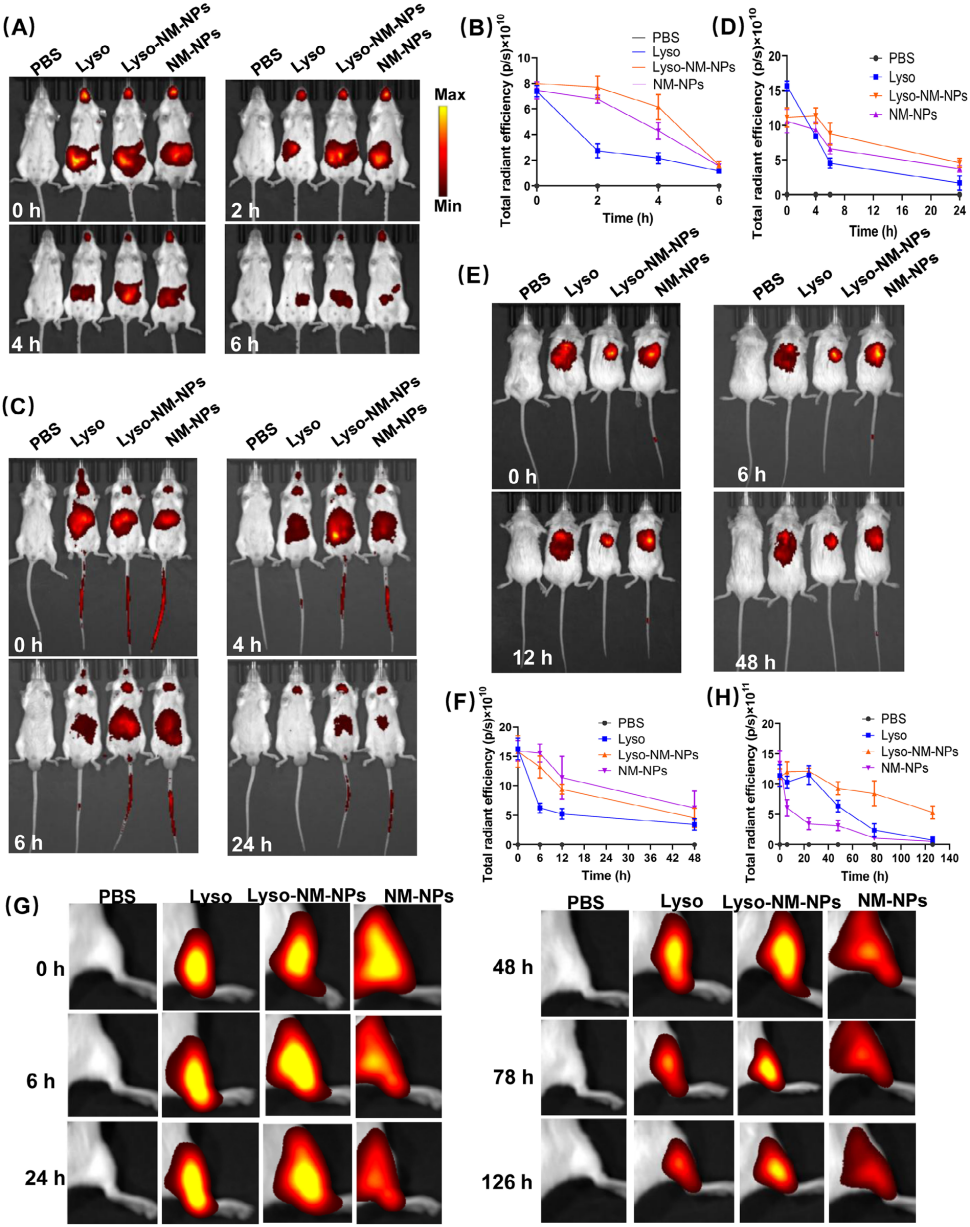
In vivo biodistribution of nanoparticles and delivery effect of loaded model proteins. Cy5.5‐labeled NM‐NPs (NM‐NPs), Cy5.5‐labeled lysozyme loaded on NM‐NPs (Lyso‐NM‐NPs, Cy5.5‐labeled Lyso), or Cy5.5‐labeled free lysozyme proteins (Lyso) were administered to mice by different forms (oral/intravenously/subcutaneous/intramuscular). (A and B) In vivo fluorescence imaging and quantitation of fluorescence intensity at different time points after oral administration. (C and D) In vivo fluorescence imaging and quantitation of fluorescence intensity at different time points after tail vein injection. (E and F) In vivo fluorescence imaging and quantitation of fluorescence intensity at different time points after subcutaneous administration. (G and H) In vivo fluorescence imaging and quantitation of fluorescence intensity at different time points after intramuscular injection. From left to right, the groups are control (PBS), Lyso, Lyso‐NM‐NPs, and NM‐NPs. Data are shown as the mean ± SD (*n* = 3).

After oral administration of the different formulations, all the mice presented strong fluorescence signals (Figures 3A and B), indicating the mice are successfully administrated. After a 2‐h period, only weak fluorescence was detected in the Lyso group, whereas strong signals were still present in the abdominal region for both the NM‐NPs and Lyso‐NM‐NPs groups even until the 4‐h mark. For the i.v. route, the mice were administered via tail vein injection. As illustrated in Figures 3C and D, the fluorescent profile showed a rapid distribution from the plasma within the first 6 h, which dropped below the limit of quantification at 24 h for the Lyso group. However, fluorescent signals in the NM‐NPs and Lyso‐NM‐NPs group were still detectable in the abdominal region even at 24 h after administration. The delivery effect of lysozyme loading on NPs over time following s.c. administration is presented in Figures 3E and F. After 6 h of injection, weak fluorescence was detected in the Lyso group, whereas strong fluorescence intensities of Lyso‐NM‐NPs and NM‐NPs were maintained at 12 h postinjection. For i.m. administrations in mice models, the fluorescence intensity of Lyso‐NM‐NPs was significantly higher and of longer duration at the injection site compared with the Lyso group (Figures 3G and H). While the fluorescence intensity declined at the injection sites at 78 h after injection in Lyso‐injected mice, strong fluorescence was still observed for Lyso‐NM‐NPs after 126 h postinjection. Interestingly, the fluorescence intensity declined more quickly for the NM‐NPs group than the other two groups for muscle administration (Figures 3G and H).

#### 2.3.2 Organ biodistribution

**FIGURE 4:**
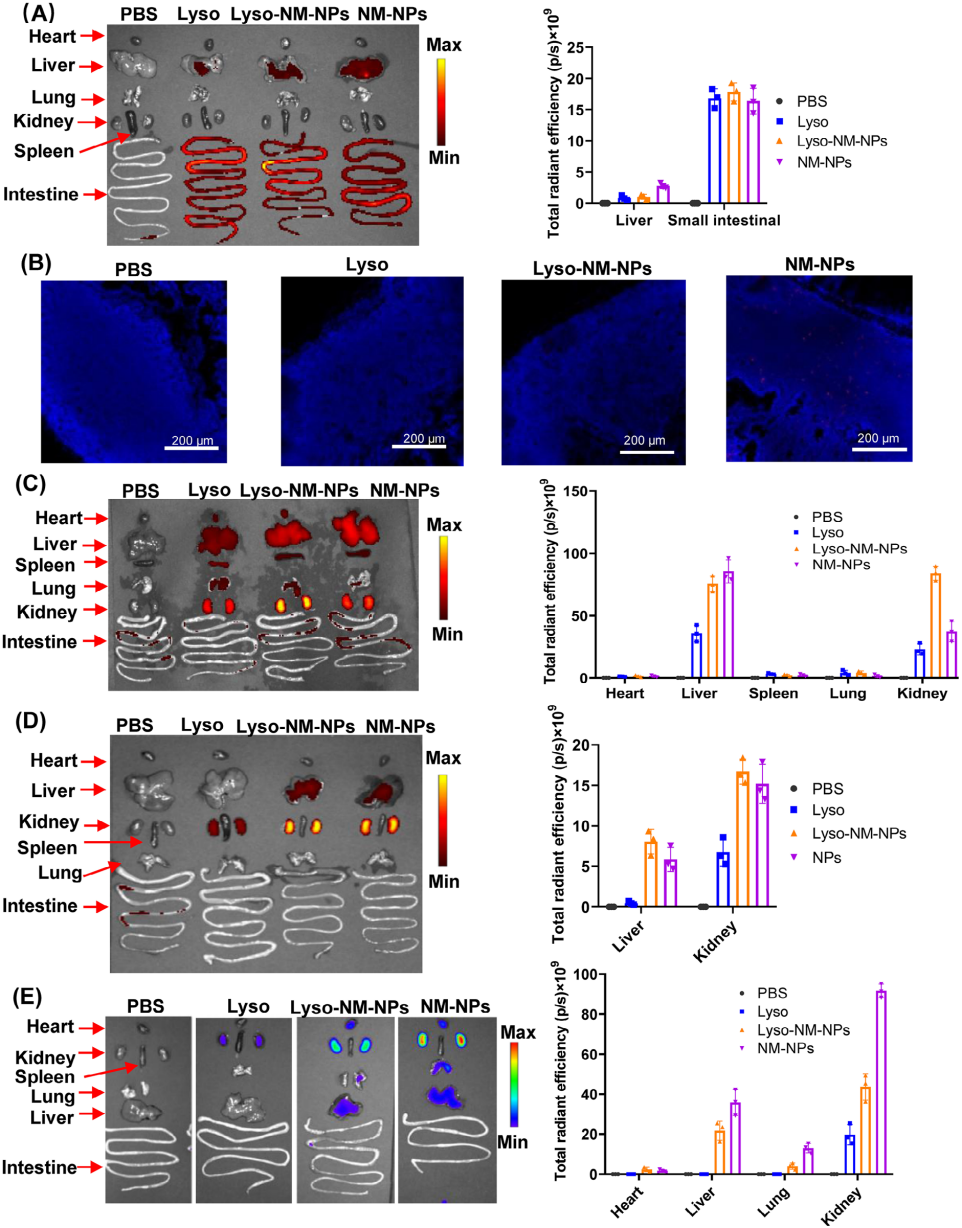
The organ biodistributions of the nanoparticles and loaded model protein. Cy5.5‐labeled different groups were administered to mice by different administration routes (oral/intravenous/subcutaneous/intramuscular). At indicated time points, the mice were sacrificed to collect the organs for ex vivo fluorescence. (A) Ex vivo fluorescence imaging (left) and quantitation of fluorescence intensity (right) of different organs after 12 h oral administration. (B) Biodistribution in Peyer's patches observed by fluorescence confocal microscope. (C) Ex vivo fluorescence imaging (left) and quantitation of fluorescence intensity (right) of different organs after 24 h tail vein injection. (D) Ex vivo fluorescence imaging (left) and quantitation of fluorescence intensity (right) of different organs after 48 h of subcutaneous administration. (E) Ex vivo fluorescence imaging (left) and quantitation of fluorescence intensity (right) of different organs after 126 h intramuscular administration. Data are shown as the mean ± SD (*n* = 3).

To further characterize the tissue distribution of protein and NPs, the mice were sacrificed and organs were collected for ex vivo fluorescence analysis at indicated time point. For oral administration, the fluorescent signals were predominantly observed in the liver and small intestine (Figure 4A). To investigate whether NM‐NPs or the loaded protein had reached the Peyer's patch (PP) dome, histological analysis of the PPs from the mice was conducted through frozen sections and observed under a fluorescence confocal microscope. Previous studies have showed that NPs smaller than 10 µm are primarily taken up by M‐cells and transported into the PPs.^27^, ^28^ When compared the differently treated groups, that is, PBS, Lyso, Lyso‐NM‐NPs, and NM‐NPs, the most prominent fluorescence was observed in the subepithelial dome for NM‐NPs (Figure 4B). These results demonstrated that NM‐NPs were resistant to the degradation of the gastrointestinal tract and efficiently reached the small intestine, where they were taken up by M‐cells and transported into the PPs. However, free and NM‐NPs delivered lysozymes were not observed in the PPs (Figure 4B), which could be explained by the quick release of the loaded lysozyme from NM‐NPs at acidic pHs and its subsequent digestion in the gastrointestinal tract. These findings indicate that NM‐NPs are not the suitable carrier for delivering protein drugs or vaccine to the PPs subepithelial dome via the per‐oral route.

For parenteral administration routes, the results indicated that the kidney was the primary target of accumulation for both formulations, followed by the liver (Figures 4C–E). Notably, the fluorescence signals of the Lyso‐NM‐NPs and NM‐NPs formulations were significantly higher than that of the free lysozyme group at different organs. Further, the biodistributions of lysozyme and NM‐NPs in the heart, spleen, and lungs after i.v. administration were also observed (Figure 4C). The NM‐NPs group had the highest fluorescence intensity in the kidney compared with the Lyso‐NM‐NPs and free lysozyme groups, which may explain the faster signal declining at the injection site after i.m. administration (Figures 3G and 4E).

These results suggest that NM‐NPs can effectively prolong the residence time of loaded proteins at injection sites and provide a sustained release effect in vivo, likely due to the depot effect and NP protection. These effects were observed for different administration routes, including i.v., s.c., and i.m. injection, but not for oral delivery, indicating NM‐NPs have potential as a protein carrier for various administration routes that could be valuable for drug and antigen delivery.

### 2.4 Cellular uptake and cross‐presentation properties of Lyso‐NM‐NPs

**FIGURE 5:**
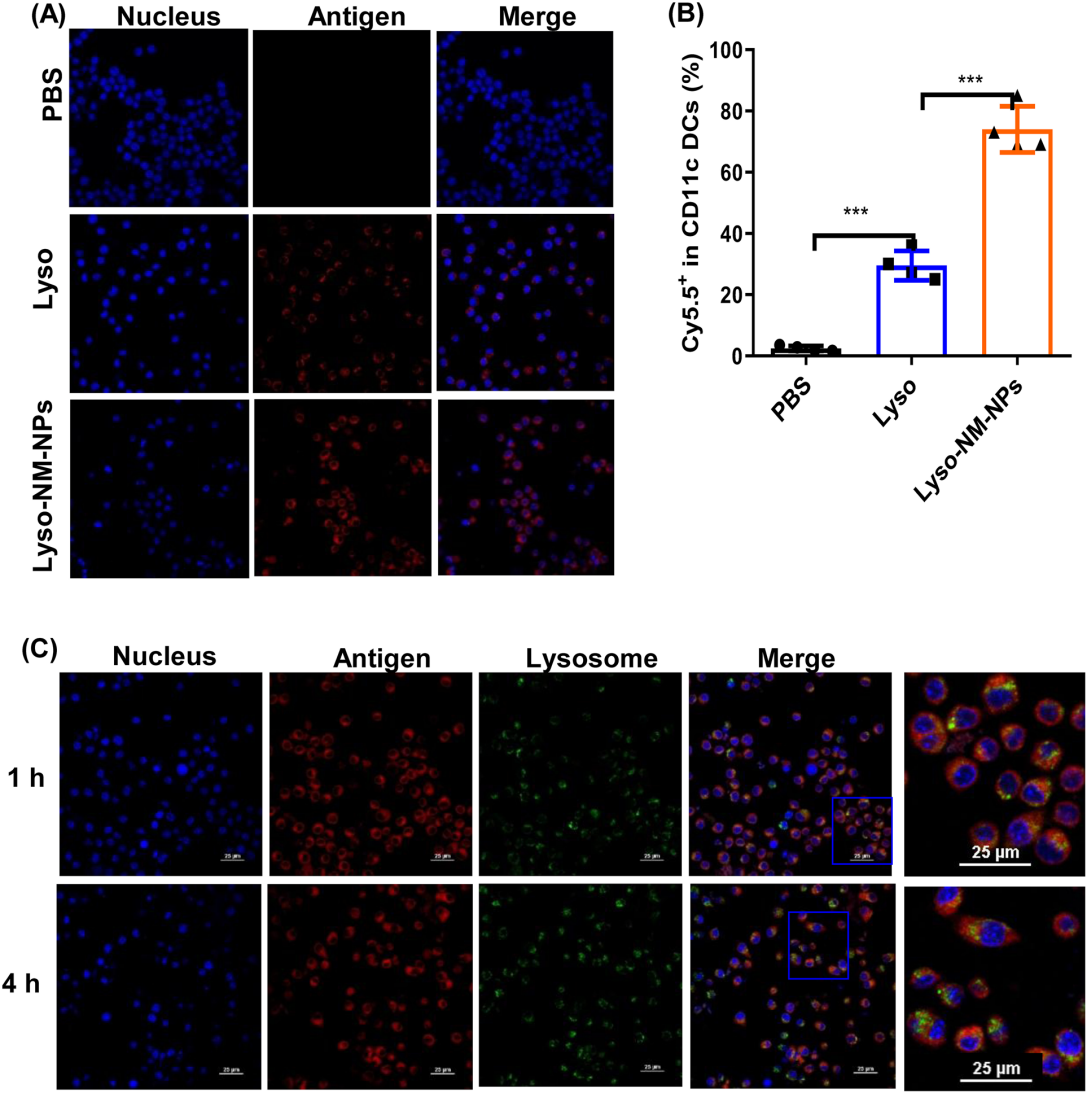
NM‐NPs enhance antigen uptake and facilitate antigen lysosomal escape in BMDCs. (A) BMDCs were cultured with Cy5.5‐labeled free lysozyme (Lyso) or lysozyme loaded on NM‐NPs (Lyso‐NM‐NPs, Cy5.5‐labeled Lyso) for 2 h. Representative confocal images of internalization antigen with different formulations. (B) Cellular uptake was quantified using flow cytometry. Data are shown as the mean ± SD (*n* = 4). Statistical analysis using one‐way analysis of variance (ANOVA): ***p < 0.001. (C) Representative confocal laser scanning images of BMDCs cultured for 1 or 4 h with Cy5.5 (red)‐labeled lysozyme loaded on NM‐NPs (LysoNM‐NPs). Nuclei were stained with DAPI (blue), lysosomes were stained with LysoTracker Green (green). The scale is 25 µm.

Considering efficient internalization by APCs is critical for immune activation, we assessed the ability of NM‐NPs with antigen loading to be taken up by bone marrow derived cells (BMDCs). We incubated BMDCs with Cy5.5‐labeled lysozyme (Lyso) or Cy5.5‐labeled lysozyme loaded on NM‐NPs (Lyso‐NM‐NPs), while cells incubated with PBS were as negative control. Confocal microscopy and flow cytometry results showed that the Lyso‐NM‐NPs group presented ∼3‐fold higher fluorescence compared with the Lyso group, suggesting NM‐NPs as a delivery carrier can significantly enhance the cellular uptake of loaded protein antigens (Figures 5A and B). We further evaluated the ability of NM‐NPs to facilitate cytoplasmic delivery of antigens after internalization into BMDCs using confocal microscopy. After 1 h incubation of BMDCs with Lyso‐NM‐NPs, yellow fluorescence resulting from the merging of antigen (red) and lysosome (green) was observed, indicating efficient cellular uptake of Lyso‐NM‐NPs. After 4 h incubation, discrete fluorescence signals for antigen and lysosomes appeared, suggesting successful escape from lysosomes and cytoplasmic delivery of the model antigen lysozyme (Figure 5C). These results together indicate that the loading of lysozyme on NM‐NPs enhanced DC internalization, lysosomal escape, and cross‐presentation.

### 2.5 Ability of Lyso‐NM‐NPs to activate immune responses and the underlying mechanism

Since the solvent‐free preparation and the processing conditions can be easily scaled‐up, and the high efficiency of loading macromolecular protein as well as the sustained release effect in vivo of different administration routes, the MiSp‐based NPs are per se suitable for the loading and delivery of protein drug/vaccine. Hence, we focused on the evaluation of whether NM‐NPs could enhance immune responses to load protein vaccine in vivo through s.c. and i.m. administrations that are the primary routes for subunit protein vaccine, and lysozyme was used as a model antigen (Table S5 and Figure S2).

#### 2.5.1 NM‐NPs enhance immune responses of loaded antigen through s.c. vaccination

**FIGURE 6:**
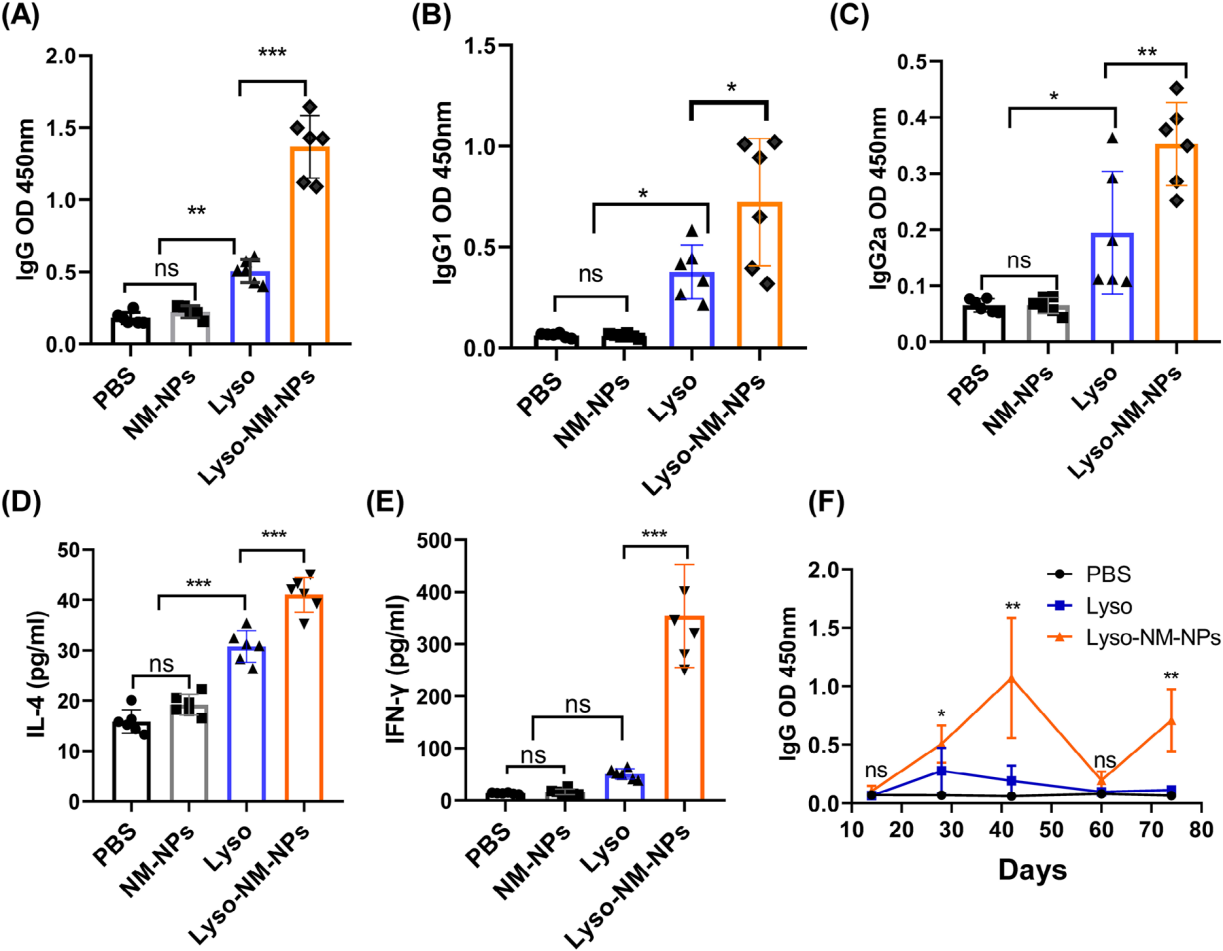
Humoral and cellular immune response in vivo. Mice were subcutaneous immunized three times on day 0, 14, and 28 with 100 µL of different vaccine formulations: PBS, NM‐NPs, Lyso, and Lyso‐NM‐NPs containing 25 µg of antigen (lysozyme). At day 35, serum from vaccinated mice was collected, and antigen specific titers of (A) IgG, (B) IgG1, and (C) IgG2a were measured using ELISA (*n* = 6). (D and E) Spleens were harvested from vaccinated mice of each group 7 days after the third immunization and concentrations of IL‐4 and IFN‐γ cytokines in the splenocytes culture medium were assessed using ELISA (*n* = 6). (F) Mice were vaccinated intramuscularly with different formulations with a single‐dose on day 0, and serum was collected on day 14, 28, 42, and 60 for measurement of antigen‐specific antibody titers of IgG by ELISA and then followed by booster immunization on day 60 to detect IgG levels on day 74 (*n* = 5). Data are shown as the mean ± SD. Statistical analysis via one‐way analysis of variance (ANOVA): *p < 0.05, **p < 0.01, ***p < 0.001; ns, not significant.

First, mice were subcutaneously vaccinated with soluble antigen alone (Lyso) or antigen loaded on NM‐NPs (Lyso‐NM‐NPs) three times on day 0, 14, and 28, with both groups receiving an equivalent amount of lysozyme, and sacrificed on day 35 and blood was collected for lysozyme‐specific antibody measurements by enzyme‐linked immunosorbent assay (ELISA). The results revealed that levels of total specific immunoglobulin G (IgG), IgG1, and IgG2a in the Lyso‐NM‐NPs‐treated group were significantly higher compared with the Lyso group (Figures 6A–C). The predominant antibody subtype induced by Lyso‐NM‐NPs vaccination was IgG1, followed by IgG2a, suggesting a potent Th2 and Th1 immune response. These results indicate that the implementation of NM‐NPs as a carrier for protein antigen is able to enhance immune responses in vivo. To further investigate the cytokine profiles, splenocytes were harvested from vaccinated mice and restimulated ex vivo with lysozyme. The levels of endogenous cytokines interferon gamma (IFN‐γ) and interleukin‐4 (IL‐4), indicative of Th1 and Th2 responses, respectively, in the supernatant were analyzed by ELISA. Strikingly, splenocytes from mice immunized with antigen loaded NM‐NPs produced much higher levels of IFN‐γ and IL‐4 cytokines than those injected with the antigen alone (Figures 6D and E). These results suggest that the NM‐NPs loading formulation can lead to a stronger mix of Th1 and Th2 immune responses.

#### 2.5.2 NM‐NPs enhance long‐term immune responses of loaded antigen through i.m. vaccination

As the obviously prolonged persistence of antigen loaded on NM‐NPs at injection sites (Figure 3G), we further investigated whether the Lyso‐NM‐NPs could induce a long‐term immune response after a single‐dose i.m. immunization. Mice were immunized intramuscularly with different formulations on day 0, and serum was collected on day 14, 28, 42, and 60 for measurement of antigen‐specific antibody titers of IgG by ELISA (Figure 6F). Results showed that Lyso‐NM‐NPs‐immunized mice exhibited higher total IgG titers than Lyso‐treated mice, peaking at 42 days postimmunization and declining thereafter, while the Lyso‐treated group showed the highest IgG level at 28 days postimmunization before declining. On days 28 and 42 postvaccination, the total IgG titers of Lyso‐NM‐NPs‐treated mice were ∼2 and ∼6‐fold, respectively, higher than those of the antigen alone‐immunized group. Upon a boost immunization on day 60, blood was collected on day 74 and lysozyme‐specific antibody titers were evaluated. The results showed that the Lyso‐NM‐NPs‐treated mice produced a fast and higher antibody response than Lyso‐treated mice, suggesting i.m. immunization with Lyso‐NM‐NPs efficiently induced long‐lasting systemic antibody responses.

These results suggested that lysozyme encapsulated in NM‐NPs is able to significantly increase the immunogenicity of lysozyme, and this enhancement depends on NM‐NPs delivery.

#### 2.5.3 Underlying mechanism

**FIGURE 7:**
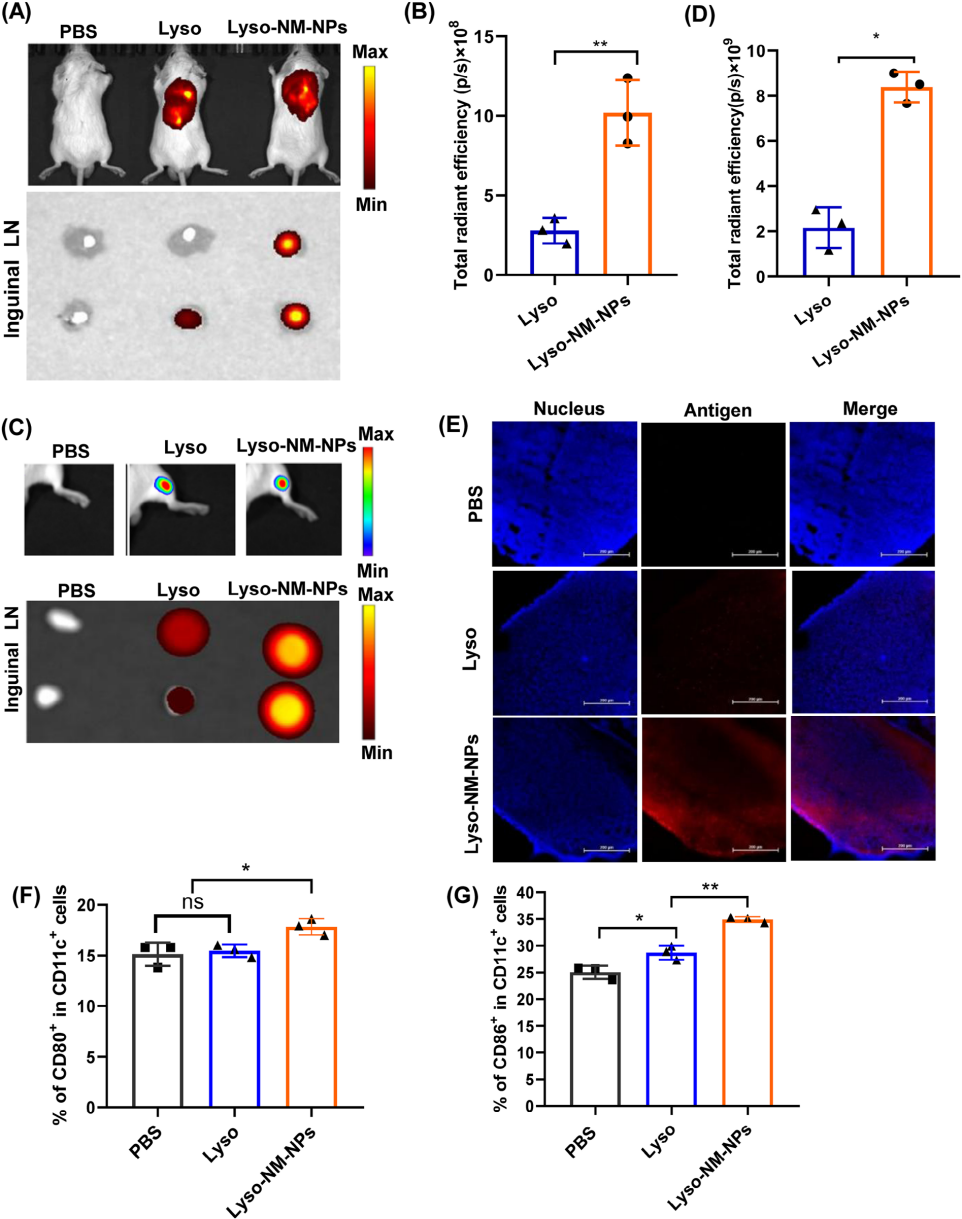
Antigen transport into draining lymph nodes and induction of dendritic cell maturation. (A and C) Fluorescence images of Cy5.5‐labeled lysozyme at injection site at time 0 and in isolated draining lymph nodes at 24 h after subcutaneously injection (A) or intramuscular injection (C). (B and D) Quantitation of fluorescence intensity in draining lymph nodes after subcutaneously injection (B) or intramuscular injection (D) (*n* = 3). (E) Antigen level in draining lymph nodes determined by immunofluorescence assay. Scale bars, 200 µm. (F and G) Relative proportions of cells positive for CD80 and CD86 surface markers were measured using flow cytometry. The data are presented as mean ± SD (*n* = 3). Statistical analysis using two‐tailed Student's *t*‐test for two groups compare, while one‐way ANOVA followed by Tukey's multiple comparison test was used for comparisons among multiple groups. *p < 0.05; **p < 0.01, ns, not significant.

To unravel the mechanisms of NM‐NPs to enhance immune responses, the draining lymph nodes were collected for ex vivo fluorescence analysis after 24 h injection. Fluorescence images demonstrated that the fluorescence intensity of Lyso‐NM‐NPs in the lymph nodes was ∼7‐fold higher (for s.c. vaccination; Figures 7A and B) or 5‐fold higher (for i.m. vaccination; Figures 7C and D) than that of Lyso, which was further confirmed by confocal imaging of lymph node slices (Figure 7E). These findings suggest that Lyso‐NM‐NPs not only provides sustained antigen exposure but also facilitates efficient antigen uptake by APCs, ultimately leading to enhanced immune responses. As the NP significantly prolongs the residence time of the model antigen at the injection site and significantly affects antigen transport to the draining lymph nodes over time, we next analyzed the extent of DC maturation in the draining lymph nodes by determining the expression of costimulatory molecules CD80 and CD86. The results showed that a significantly higher expression of CD80/86 was observed for Lyso‐NM‐NPs‐treated group compared with free lysozyme group at 48 h after s.c. injection (Figures 7F and G and S3), indicating Lyso‐NM‐NPs possesses potent immunostimulatory ability.

Collectively, these findings offer compelling evidence that the NM‐NPs loading approach significantly augments systemic immune responses, whether administered subcutaneously or intramuscularly. This underscores the remarkable adaptability of NM‐NPs as delivery vehicles across diverse administration routes.

### 2.6 Cytotoxicity and biosafety of NM‐NPs

**FIGURE 8:**
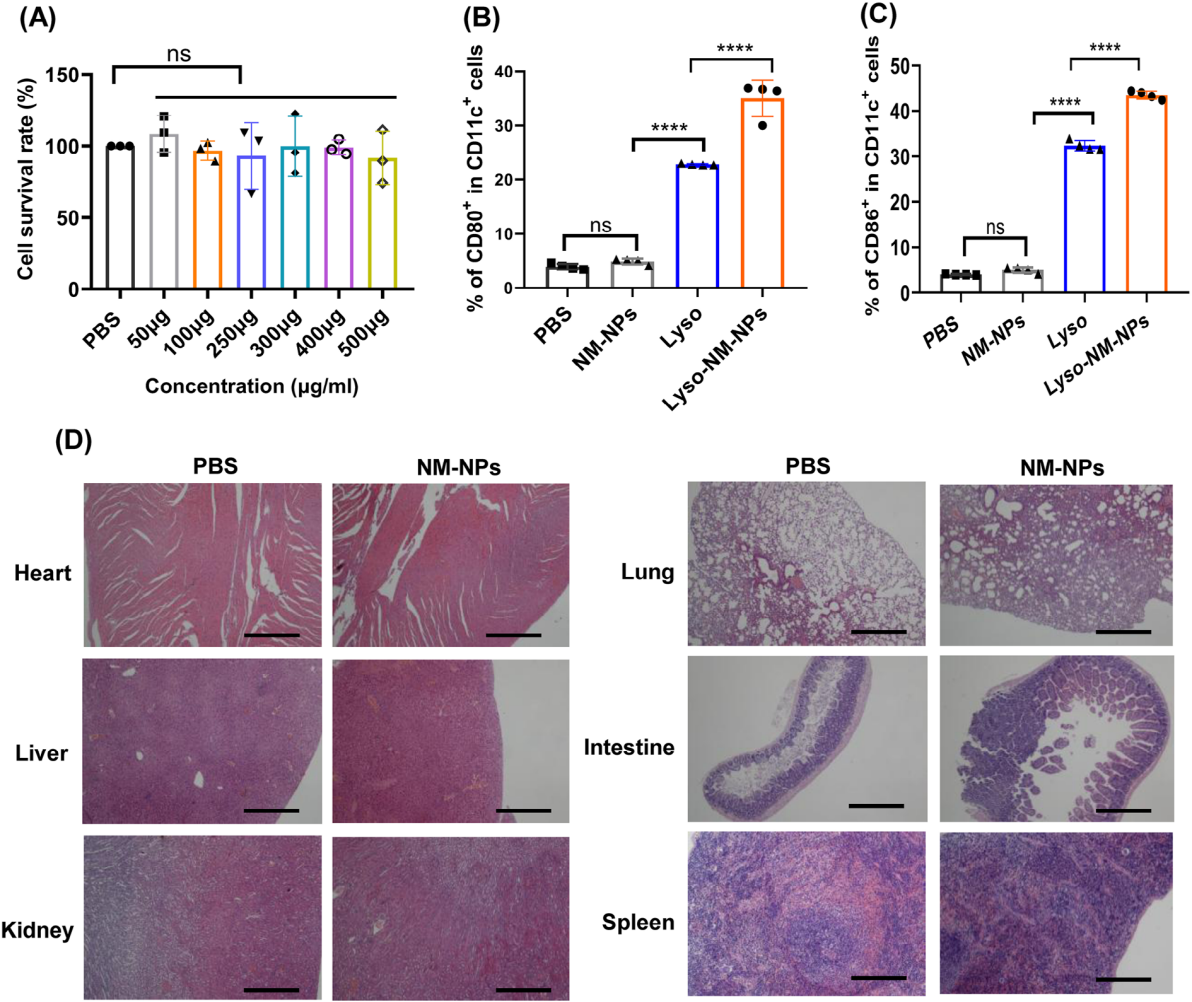
Cytotoxicity and biocompatibility in vitro/vivo of NM‐NPs. (A) Relative viability of BMDC cells in culture exposed to different concentration of NM‐NPs was evaluated using the CCK‐8 Cell Proliferation Assay Kit (*n* = 3). (B and C) Flow cytometry analysis the percentage of CD80 (B) and CD86 (C) expression on BMDCs with different treatments (*n* = 4). (D) Histological section of mice heart, liver, kidney, spleen, lung, and intestine after three times repeated intravenous administrations of NM‐NPs (250 µg/mice) to the control group administered with PBS alone. The sections were stained with hematoxylin‐eosin and the scale bar is 50 µm. The data are presented as mean ± SD. Statistical analysis via one‐way analysis of variance (ANOVA): ****p < 0.0001, ns, not significant.

The excellent loading behavior and sustained releasing profile make the NM‐NPs as a promising candidate for protein drug/vaccine delivery. With the aim of biomedical applications, we conducted an evaluation of the cytotoxicity of NM‐NPs on APCs. Specifically, we exposed BMDCs to different concentrations of NM‐NPs (50–500 µg mL^−1^). After 24 h of exposure, no discernible signs of toxicity upon addition of different concentrations of NM‐NPs to the culture media were detected, as demonstrated by the qualitative comparison of cell density between the control group (PBS) and the NM‐NPs‐treated group (Figure 8A).

Next, we investigated whether these NPs lead to unspecific DC maturation in themselves. BMDCs were incubated with NM‐NPs for 24 h before testing the costimulatory factors on the cell surface (CD80 and CD86). The result showed that none of the particle in themselves increased the density of the costimulatory factors, while Lyso and Lyso‐NM‐NPs significantly induced the BMDCs maturation through upregulation of CD80 and CD86 (Figures 8B and C and S4). Thus, the spider silk NPs derived from NM protein did not show unspecific immune activation in vitro.

To get some insights into the biocompatibility, the biosafety of the NM‐NPs in vivo were assessed after multiple i.v. administration to mice. We conducted a histological examination of tissues from the treated mice. Notably, repeated administration of NM‐NPs did not induce any signs of inflammation or fibrosis upon microscopic observation of the heart, liver, spleen, lungs, kidney, and intestine (Figure 8D). The excellent biocompatibility and biosafety renders the possibility to implement NM‐NPs as a delivery protein drug/vaccine carrier in vivo.

## 3 DISCUSSION

**FIGURE 9:**
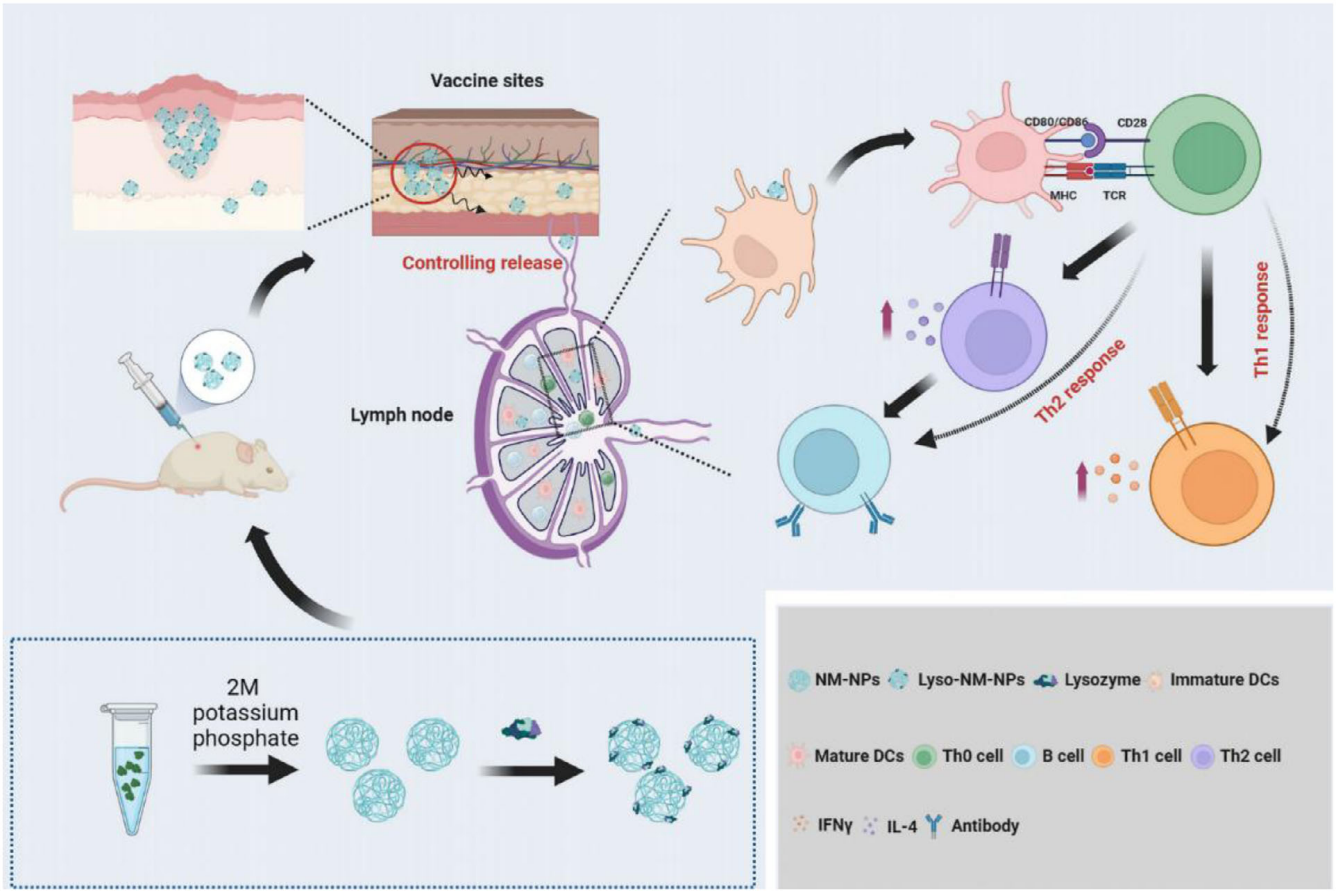
Spider‐silk based nanoparticles as a suitable carrier for protein antigen induce a robust immune and cellular responses by subcutaneous immunization. These nanoparticles, based on MiSp, significantly enhance antigen‐specific immune responses, through mechanisms involving antigen‐depot effects at the injection site, efficient uptake by dendritic cells as well as internalization by lymph nodes, promoting maturation and cross‐presentation.

NPs based on natural polymers like spidroin have emerged as promising delivery vehicles due to their biocompatibility and biodegradability, which allow them to control the release of loaded agents and create a sustained release pattern.^29^, ^30^, ^31^, ^32^, ^33^, ^34^ In this study, we demonstrate that the applicability of MiSp‐based NPs, made from a customized spidroin covering the NT domain and a repetitive region, as an efficient protein delivery system for macromolecular protein subunit vaccines with inducing enhanced systemic immune responses in vivo (Figure 9).

NP properties, such as size and shape, can affect cellular and tissue uptake as well as immune responses.^35^, ^36^, ^37^ Previous studies have shown that large NPs (200−2000 nm) can be effectively taken up by the migratory DCs and peripheral APCs at the injection site to transport to lymph nodes for production more humoral responses, while small ones (20–200 nm) is able to drain freely into lymph nodes and are presented to resident DCs within hours.^38^, ^39^, ^40^ The shape of NPs also affects their biodistribution and uptake efficiency by APCs.^41^ For example, spherical gold NPs were effectively taken up by APCs compared with rod‐shaped counterparts. The MiSp‐based NM‐NPs are with a size of ∼200 nm and a spherical morphology (Figure 1), after loaded with protein, the Lyso‐NM‐NPs can increase to approximately 500 nm in diameter with maintaining the spherical morphology (Figure 2B). This size is considered as an interesting property for an antigen depot, providing a controlled release formulation and guaranteeing efficient delivery of antigens to APCs, which is suitable as carrier for delivering antigens.

Apart from sphere properties, another crucial factor is the kinetics of antigen exposure to the immune system.^42^ NM‐NPs might form complexes with positively charged molecules likely based on electrostatic interactions due to their negative charge at neural pH,^43^ resulting in a loading efficiency of lysozyme on NM‐NPs of approximately 100%, similar to the binding efficiency of lysozyme to NPs derived from dragline silk protein via electrostatic interactions.^10^ The release assay of the antigen from NM‐NPs indicates a slow release at neural pH, which may act as an antigen depot for a better presentation of antigens to APCs. In contrast, ∼90% of lysozyme released immediately in buffer pH 2 or 4 (Figure 2E), consistent with the results that lysozyme loaded on pH‐responsive Lyso‐NM‐NPs taken up by APCs could induce lysosomal swelling, leading to antigen release into the cytoplasm.^44^ Endo‐lysosomal escape of antigens can greatly promote cross‐presentation, in other words, the escape antigen could be processed through MHC‐I pathway, as cross‐presentation is essential for the initiation of CD8^+^ T cell responses for elimination of pathogen‐infected cells, especially CTLs.^45^ Following antigen exposure, maturation and activation of DCs is a prerequisite of effective antigen presentation and subsequent T cell activation. An ideal vaccine delivery system should activate CD4^+^ T cells differentiation into subsets of helper T cells such as Th1 and Th2 cells, which are known to promote the activation of CTLs or B cells by producing antibodies.^44^, ^46^ In our study, loaded antigen on NM‐NPs not only provided long‐term antigen retention at the injection site but also achieved sustained antigen transport into the draining lymph node, which induced higher DC activation in the draining lymph nodes (Figures 3 and 7). Additionally, Lyso‐NM‐NPs‐immunized mice induced a powerful immune and cellular responses compared with free lysozyme (Figure 6). The aforementioned results can explain why and how the nanovaccine can elicit more powerful antigen‐specific immune responses than protein vaccine alone. NM‐NPs as carriers can control the release of antigens at the injection site (depot effect), protect the antigen from degradation and clearance, enhance antigen uptake by APCs, promote DC maturation and facilitate antigen loading onto MHC molecules class I and/or II, inducing both Th1 (cellular) and Th2 (humoral) immune responses. A mixed Th1/Th2 immune response is critical for developing a vaccine against pathogens capable of creating extracellular and intracellular reservoirs.^47^ Meanwhile, a single i.m. immunization of Lyso‐NM‐NPs leads to a sustain‐specific serum IgG production up to 60 days (Figure 6F), indicated that a continuous and slow release of antigen from the particles causes prolonged contact of antigens with the APCs, thus improving the immune responses and facilitating a long‐term memory T cell response.^48^, ^49^ In our repeated administration studies, we observed no signs of histological damage or inflammation in tissues (Figure 8D). Also, the spider silk NPs did not show unspecific immune activation (Figures 8B and C). These findings provide promising evidence for the safe use of this material in biological systems and suggest its potential for successful biomedical applications.

## 4 CONCLUSION

In the current study, we explored the potential use of spider MiSp‐derived NM‐NPs as a nanocarrier for protein antigens/vaccine. Specifically, we investigated the loading of lysozyme, a model antigen, onto NM‐NPs and found that the antigen was efficiently loaded and exhibited a sustained release profile. Loading onto NM‐NPs facilitated efficient endocytosis by BMDCs and led to significant increases in specific IgG, IgG1, and IgG2a levels. The mechanisms underlying how NM‐NPs act as a vaccine carrier to promote antigen‐specific immune responses are multifaceted (Figure 9). First, the NPs establish an antigen depot at the injection site, allowing for long‐term antigen persistence and sustained uptake by APCs in the draining lymph node. This provides a persistent supply of antigen to the immune system. Second, sufficient initial antigen exposure and prolonged antigen persistence lead to DC maturation in the draining lymph nodes. Thirdly, lysozyme loaded on pH‐sensitive NM‐NPs taken up by APCs induces lysosomal swelling and antigen escape, promoting the induction of cross‐presentation.

The strength of this study demonstrates the effective use of protein NPs, derived from biocompatible MiSp,^20^, ^50^ for protein delivery, including the loading/releasing profile and the ability to enhance specific immune responses. The clinical potential of these NPs is evident in their high lysozyme loading efficiency and sustained release capabilities, heralding advances in therapeutic protein and vaccine delivery. Nevertheless, the use of lysozyme—a model protein rather than a pathogen‐specific vaccine—marks a limitation of this study, suggesting the need for further investigation into the comprehensive application of this NP‐based delivery system in addressing real‐world infectious diseases within translational medicine.

## 5 MATERIALS AND METHODS

### 5.1 Protein preparation

A gene fragment encoding the 161‐aa N‐terminal sequence followed by 261‐aa repetitive sequence of *A. ventricosus* MiSp (designated as NM) was synthesized and inserted into pET‐32a plasmid within *Nde*I and *Xho*I restriction sites (pET‐NM) as described before.^51^ For protein preparation, the cells were lysed and the pellets (NM‐IBs) were collected for subsequent solubilization study (S1.1, Supporting Information). Three methods of solubilization were applied to obtain NM protein according to the previous study (S1.2, Supporting Information).^51^, ^52^ Supernatants (10 µL) were analyzed by SDS‐PAGE to check the quality of the protein. Protein concentration was estimated by Micro BCA Protein Assay Kit (Thermo Scientific, Rockford, USA). The secondary structures of soluble proteins were measure using CD spectroscopy (S1.3, Supporting information)

### 5.2 Particles preparation and characterization

The NM‐NPs were prepared by salting out with a potassium phosphate solution as described previously.^15^, ^16^, ^51^ The particle preparation and characterization was described in S1.4, Supporting Information.

### 5.3 In vitro protein loading and releasing

A model protein solution was prepared by dissolving 0.1 mg lyophilized lysozyme (Amresco; lysozyme chloride formed from chicken egg white) in 10 mM phosphate buffer (PBS) at pH 7.4 (100 µg/mL). Then, 1 mg of NM‐NPs was added in the solution and incubation at 4°C for 2 h. The mixture was centrifuged and the supernatant was analyzed for residual protein content using the Micro BCA Protein Assay Kit (Thermo Scientific). To further demonstrate the binding of lysozyme on NM‐NPs, released lysozyme from loaded NM‐NPs was analyzed by SDS‐PAGE (S1.5, Supporting Information). Encapsulation efficiency and loading were determined using equations (1) and (2), respectively.

(1) $$\begin{array}{ccc} & & encapsulation\;efficiency\;\left(\%\right)\\ & & =\frac{\mathrm{total}\;\mathrm{amount}\;\mathrm{of}\;\mathrm{lysozyme}-\mathrm{free}\;\mathrm{lysozyme}}{\mathrm{total}\;\mathrm{amount}\;\mathrm{of}\;\mathrm{lysozyme}\;\mathrm{added}}\times 100\% \end{array}$$

(2) $$\begin{array}{c} \mathrm{load}\mathrm{ing}\left(w/w\%\right)=\frac{\;\mathrm{weight}\;\mathrm{of}\;\mathrm{lysozyme}\;\mathrm{in}\;\mathrm{particles}}{\mathrm{weight}\;\mathrm{of}\;\mathrm{nanoparticles}}\times 100\% \end{array}$$

### 5.4 Mice

All experimental protocols and animal analyses were conducted according to the Guide for the Care and Use of Medical Laboratory Animals (Ministry of Health, China, 1998) and with the ethical approval the guide of Soochow University. BALB/c mice were purchased from the Experimental Animal Center of Chinese Academy of Science (Shanghai, China) and used at 6−8 weeks of age.

### 5.5 Tissue biodistribution in vivo

For different administration routes (oral/i.v./s.c./i.m.), mice were treated with 100 µL of PBS containing 100 µg of Cy5.5‐labeled NM‐NPs or 100 µg of Cy5.5‐labeled free lysozyme (Lyso) or 100 µg of Cy5.5‐labeled lysozyme loaded on NM‐NPs (Lyso‐NM‐NPs), and PBS as a negative control (S1.8, Supporting Information). Detailed descriptions of different administration routes was shown in supporting information (S1.9). At the indicated time points, mice were scanned using the PerkinElmer's in vivo imaging system (IVIS Spectrum, Massachusetts, USA) at an excitation of 673 nm and emission of 692 nm. A background scan was taken with administration of PBS, and this provided the thresholding for adjusting the images collected at later time points. Then at indicated time for different administration forms, the heart, kidney, liver, lung, spleen, and small intestines were collected for imaging observation. The fluorescence intensity was quantified by using Living Image 4.0 software.

For immunofluorescence assay, the fresh isolated organs were embedded with OCT (optimal cutting temperature compound). Five‐micrometer frozen sections were prepared and dropped Hoechst 33342 (Beyotime, China) onto the slide and cover the tissue adequately. The images were obtained using a confocal microscope (Nikon, Japan).

### 5.6 Cellular uptake and lysosomal escape of antigens in vitro

BMDCs were generated as previously described (S1.10, Supporting Information).^53^ For cellular uptake, BMDCs were seeded in 24‐well plates at a density of 1 × 10^5^ cells/well and incubated in complete medium for 24 h before use, and then the medium in each well was replaced with medium containing 10 µg/mL (final concentration) of Cy5.5‐labeled lysozyme loaded on NM‐NPs (Lyso‐NM‐NPs) or Cy5.5‐labeled free lysozyme (Lyso). Cells were incubated for 2 h at 37°C and the cell pellet was washed three times with PBS. Then the cells were observed using confocal fluorescence microscope (Nikon) and analyzed using Canto II & Calibur flow cytometry (Beckman Coulter, USA).

The lysosomal escape ability of antigens was evaluated on BMDCs with a confocal laser scanning microscope (Nikon). BMDCs seeded in confocal dishes were incubated with NM‐NPs containing a concentration of 10 µg/mL Cy5.5‐labeled lysozyme for 1 h and 4 h at 37°C. Cells were then stained with LysoTracker Green and DAPI to visualize the lysosomes and nucleus.

### 5.7 Animal immunization and immune responses

As shown in Table S5 and Figure S2, male BALB/c mice aged 6−8 weeks were randomly divided into four groups and immunized with 100 µL of different vaccine formulations containing 25 µg of antigen (lysozyme). For s.c. immunization (six animals per group), mice were immunized three times at 2‐week intervals and blood samples were collected for later analysis at 7 days after the third immunization. For i.m. vaccination (five animals per group), mice were immunized with different vaccines on day 0 and a booster immunization on day 60 (50 µL/hind leg). Then, serum was collected on day 14, 28, 42, 60, and 74 for measurement of antigen‐specific antibody titers of IgG by ELISA (S1.11, Supporting Information). For determination of cytokine production, IFN‐γ and IL‐4 were measured by the ELISA kit (eBioscience, USA) according to the manufacturer's instructions (S1.12, Supporting Information).

### 5.8 Uptake of lysozyme and induction of DC maturation in vivo

The mice were grouped with three mice per group and s.c./i.m. given 100 µL of PBS containing 25 µg Cy5.5‐labeled lysozyme in different formulations (Lyso and Lyso‐NM‐NPs). At 24 h after administration, mice were sacrificed by neck dislocation and isolated the draining lymph nodes. For imaging observation, the isolated lymph nodes were scanned using the in vivo imaging system (IVIS Spectrum) at an excitation of 673 nm and emission of 692 nm. For the induction of DC maturation, cells were collected from draining lymph nodes, and expression of CD80 and CD86 on CD11c^+^ DCs was determined by flow cytometry (Beckman Coulter) (S1.13, Supporting Information).

### 5.9 NM‐NPs biosafety assay

The biosafety assay of NM‐NPs including cell cytotoxicity assay (S1.14, Supporting Information), tolerance in mice (S1.15, Supporting Information), and unspecific immune responses assay (S1.16, Supporting Information).

### 5.10 Statistical analysis

The results presented in this study are expressed as means ± SD. Statistical analyses were conducted using GraphPad Prism 9.0 software (San Diego, CA, USA). To compare two groups, an unpaired, two‐tailed Student's *t*‐test was performed, while one‐way ANOVA followed by Tukey's multiple comparison test was used for comparisons among multiple groups. Statistical significance was expressed as follows: *p < 0.05, **p < 0.01, ***p < 0.001, and ns, no significance.

## AUTHOR CONTRIBUTIONS

H. Y., L. L., G. W., Y. L., and X. Q. performed the experiments. S. X., X. Q., and G. C. conceived and supervised this study. G. C. and X. Q. analyzed the data. G. C. and X. Q. wrote the manuscript. All authors have read and approved the final manuscript.

## CONFLICT OF INTEREST STATEMENT

The authors declare no conflict of interest.

## ETHICS STATEMENT

All animal experimental protocols were approved by the Soochow University Animal Care Committee and research protocols were conducted in accordance with the animal behavioral guidelines, using approved protocols from the institutional animal care committee (#202210A0142).

## Supporting information

Supporting Information

## Data Availability

All data and materials related to this paper are available upon request.
