# Supplementary material for: Spider minor ampullate silk protein nanoparticles: an effective protein delivery system capable of enhancing systemic immune responses
Source: MedComm (2020). 2024 Jun 15;5(7):e573. doi: 10.1002/mco2.573 (PMC11179522; doi:10.1002/mco2.573)
Supplement: Supplementary file 1 — Supporting Information [file MCO2-5-e573-s001.docx]

**Supporting information**

**Spider minor ampullate silk protein nanoparticles: an effective protein delivery system capable of enhancing systemic immune responses**

**Running title: silk nanoparticle as delivery for protein vaccine**

Hairui Yu^1#^, Gefei Chen^2#*^, Linchao Li^1#^, Guoqiang Wei^1^, Yanan Li^3^, Sidong Xiong^1*^, Xingmei Qi^1*^

^1^The Jiangsu Key Laboratory of Infection and Immunity, Institutes of Biology and Medical Sciences, Soochow University, 215123 Suzhou, China

^2^Department of Biosciences and Nutrition, Karolinska Institutet, 14 183 Huddinge, Sweden

^3^Department of Neurosurgery, Changhai Hospital, Naval Medical University, 200433 Shanghai, China

^#^These authors contributed equally to this work.

^*^Corresponding author: [xmqi@suda.edu.cn](mailto:xmqi@suda.edu.cn), [sdxiongfd@126.com](mailto:sdxiongfd@126.com) and [gefei.chen@ki.se](mailto:gefei.chen@ki.se),

**Table S1 Characterization of NM-NPs**. Data are shown as mean ± SD. Size and zeta potential were measured in triplicate by dynamic light scattering on a Zetasizer NanoZS.

| Methods | Particle size (nm) | Zeta potential (mv) | Polydispersity index |
| --- | --- | --- | --- |
| Freezing-thawing | 211.2±9.1 | −23.5±0.8 | 0.294±0.004 |
| Heating | 299.8±8.1 | −25.8±0.8 | 0.293±0.02 |
| Traditional | 355.6±7.9 | −25.6±0.2 | 0.482±0.004 |

**Table S2** Lysozyme loading on NM-NPs

| Group | Lysozyme solution  (before loading) | Lysozyme solution  (after loading) | NM-NPs | encapsulation  efficiency (%) | Loading  (w/w%) |
| --- | --- | --- | --- | --- | --- |
| 1 | 100 μg | 1.2 μg | 1000 μg | 98.8 | 9.88 |
| 2 | 100 μg | 1.5 μg | 1000 μg | 98.5 | 9.85 |
| 3 | 100 μg | 1.0 μg | 1000 μg | 99.0 | 9.90 |

**Table S3** Different administrations for *in vivo* tissue biodistribution.

| Group | NM-NPs (Blank) | Lyso-NM-NPs  (Lysozyme loaded) | Lyso (Lysozyme soluble) | PBS |
| --- | --- | --- | --- | --- |
| NPs | 100 μg (Cy5.5 labeled) | 1000 μg^1^ | 0 | 0 |
| lysozyme | 0 | 100 μg (Cy5.5 labeled) | 100 μg (Cy5.5 labeled) | 0 |
| PBS | 100 μL | 100 μL | 100 μL | 100 μL |

^1^ 1000 μg of NM-NPs encapsulation 100 μg of Cy5.5 labeled lysozyme.

For different administrations (oral/intravenously/ subcutaneous/ intramuscular), BALB/c mice (age 6- 8 weeks) were prepared and each group consisting of 3 mice, and the study was repeated two times. NM-NPs group was treated with 100 μL of PBS containing 100 μg of Cy5.5 labeled NM-NPs (1mg/mL). Lyso group was treated with 100 μL of PBS containing Cy5.5 labeled free lysozyme (1mg/mL). Lyso-NM-NPs group was treated with 100 μL of PBS containing Cy5.5 labeled lysozyme loaded on NM-NPs (10 mg/mL Lyso-NM-NPs containing 1 mg/mL lysozyme). PBS group was treated with 100 μL of PBS as a negative control.

**Table S4** The fluorescence of different NM-NPs before and after labelling of Cy5.5

|  | samples | before labeling | after loading | wash buffer |
| --- | --- | --- | --- | --- |
| NM-NPs | 1 | 2.470 | 131.4 | 10.49 |
|  | 2 | 3.050 | 129.8 | 9.907 |
|  | 3 | 2.298 | 128.5 | 12.27 |
| Cy5.5 solution | 1 | 1124 | 138.3 |  |
|  | 2 | 1160 | 149.0 |  |
|  | 3 | 1141 | 156.0 |  |

**
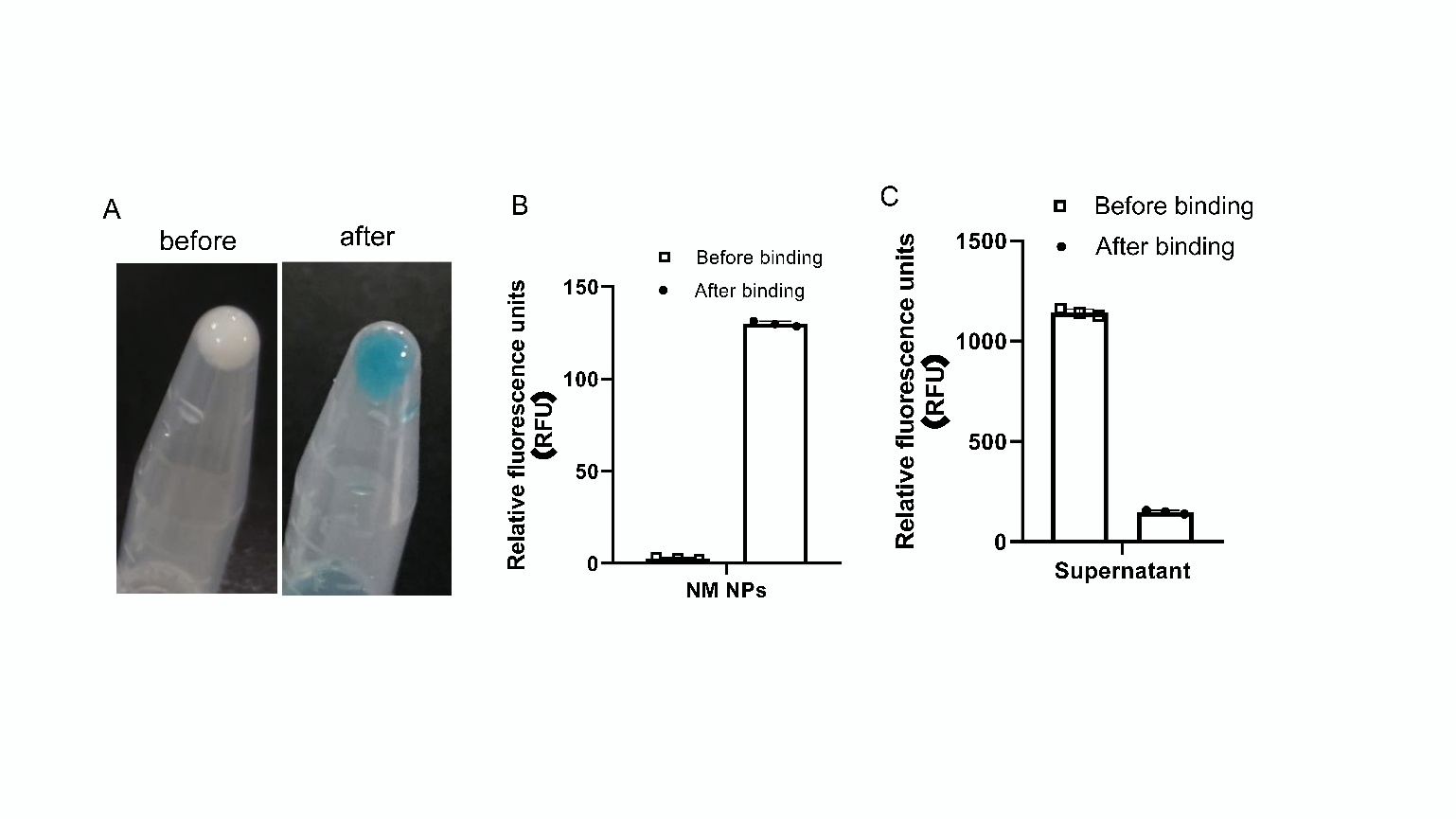
**

**Figure S1 Cy5.5 labeling of NM-NPs.** (A) The images of NM-NPs before and after binding with Cy5.5. (B) The fluorescence of NM-NPs before and after labelling with Cy5.5. (C) The fluorescence of Cy5.5 solution before and after labelling with NM-NPs.

**Table S5** Different vaccine formulations.

| Group | NM-NPs (Blank) | Lyso-NM-NPs (Lysozyme loaded) | Lyso (Lysozyme soluble) | PBS |
| --- | --- | --- | --- | --- |
| NPs | 250 μg | 250 μg^1^ | 0 | 0 |
| Ag | 0 | 25 μg | 25 μg | 0 |
| PBS | 100 μL | 100 μL | 100 μL | 100 μL |

^1^ 250 μg of NM-NPs encapsulation 25 μg of lysozyme.


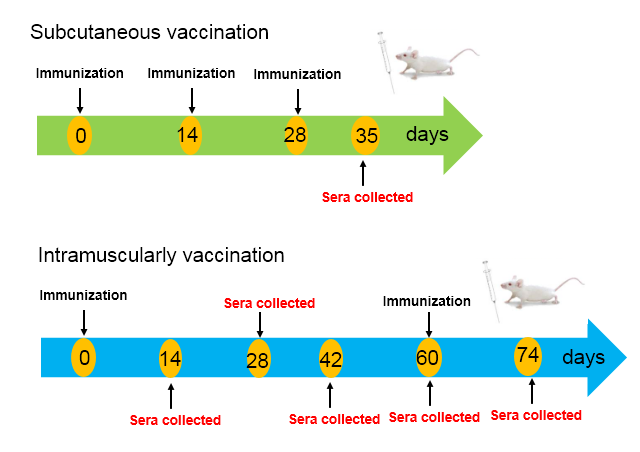


**Figure S2 Immunization scheme.** Male Balb/c mice (6-8 weeks old, *n* = 6) were immunized by subcutaneous or intramuscular injection with 100 μL of PBS containing different vaccine formulations. For subcutaneous immunization, mice were immunized 3 times at 2-week intervals (day 0, 14, and 28) injection into the dorsal flanks. At 7 days after the third immunization (day 35), blood samples were collected, and sera were isolated by centrifugation. For intramuscularly vaccination, mice were immunized with different vaccines on day 0 and a second immunization on day 60 (50 μL/hind leg, 100 μL total/mouse). Then, serum was collected on day 14, 28, 42, 60 and 74 for measurement of antigen-specific antibody.


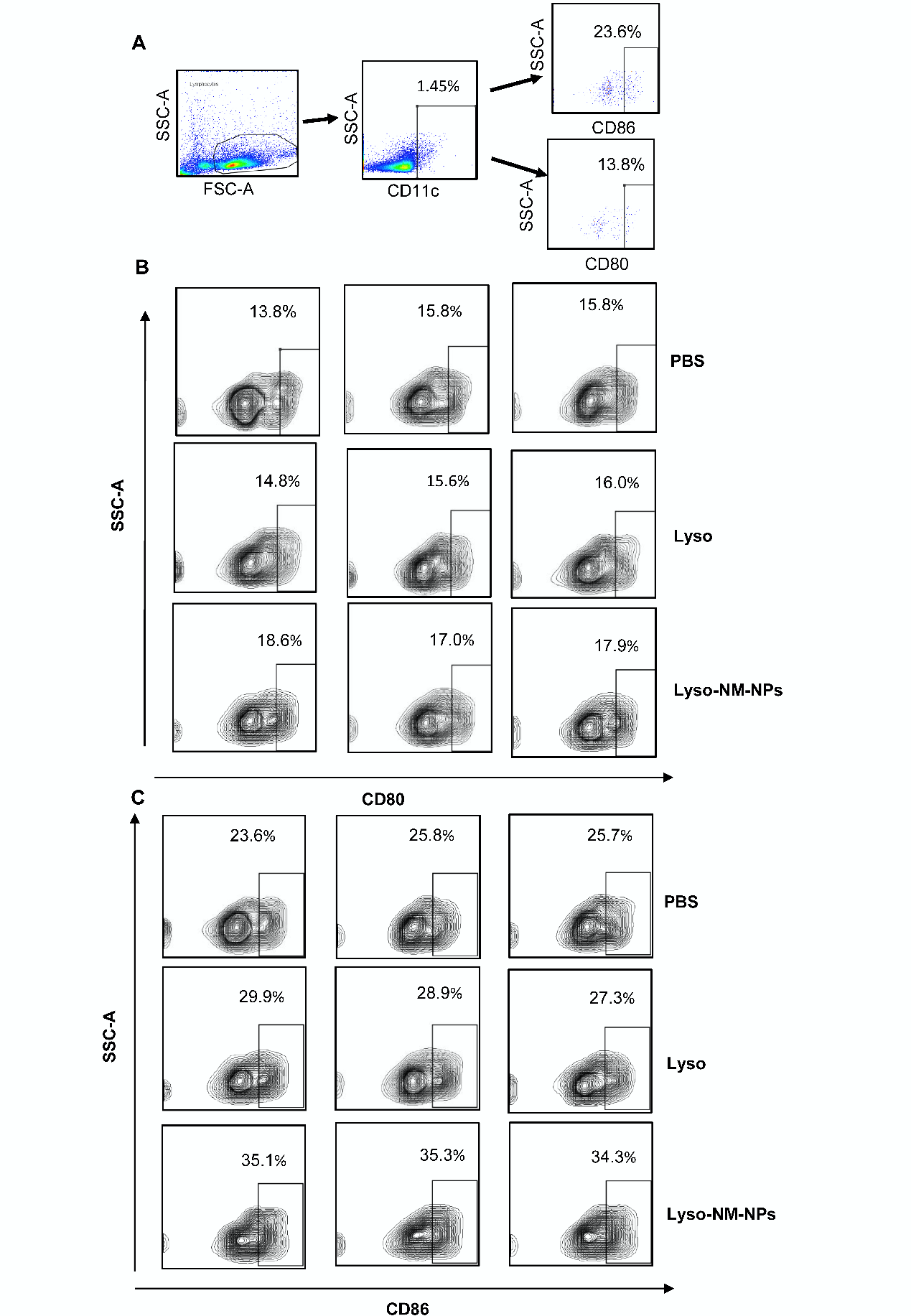


**Figure S3** (A) The gated strategy of positive regions of CD11c single positive, CD11c ^+^CD80^+^ and CD11c ^+^CD86^+^. (B) The extent of dendritic cell (CD11c ^+^) maturation in the draining lymph nodes by determining the expression of costimulatory molecules CD80 and CD86 using flow cytometry.


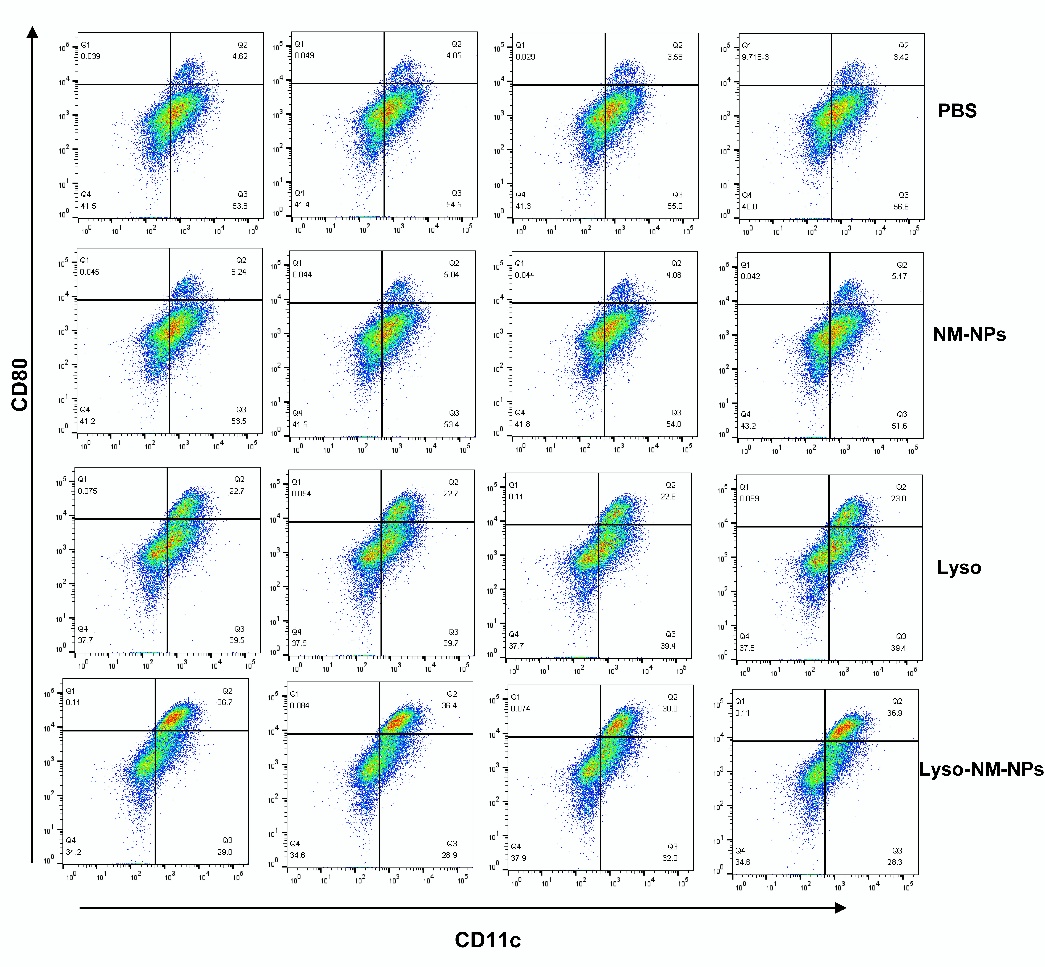


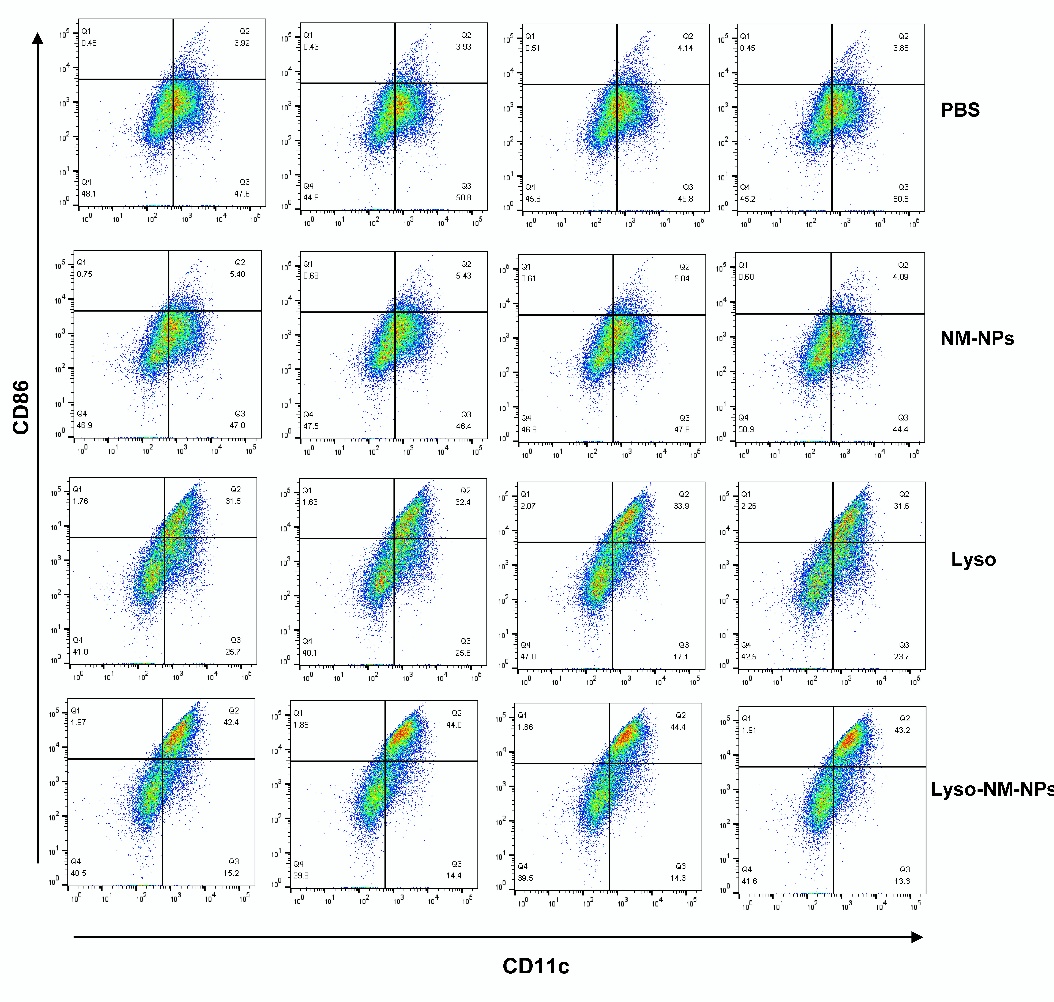


**Figure S4 Flow cytometry analysis the percentage of CD80 and CD86 expression on BMDCs with different treatments.** BMDCs were seeded on 12-well plates at a density of 1×10^6^ cells per well and incubation with free lysozyme (final lysozyme concentration was 20 μg mL^−1^), empty NM-NPs (final NM-NPs concentration was 200 μg mL^−1^), NM-NPs containing lysozyme (final NM-NPs concentration was 200 μg mL^−1^ containing lysozyme 20 μg mL^−1^) and PBS for 24 h, respectively. The BMDCs were collected and the expression of CD80 and CD86 on CD11c^+^ DCs was determined by flow cytometry.


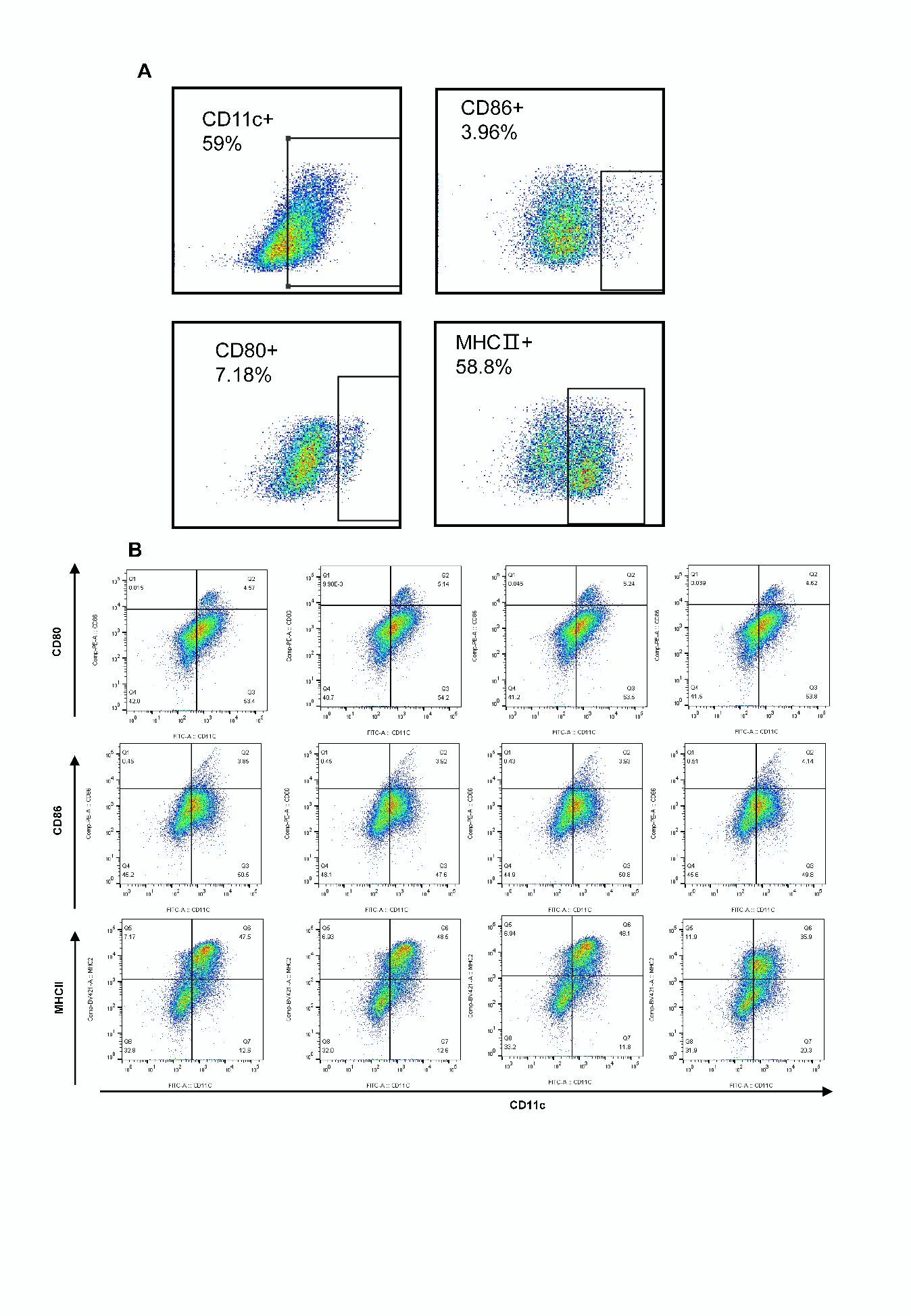


**Figure S5** Flow cytometry analysis the percentage of surface markers of CD80, CD86 and MHCII expression on BMDCs after cell culture.

**Table S6** The evaluation of LPS in the protein samples.

| Sample | 1 | 2 | 3 | 4 |
| --- | --- | --- | --- | --- |
| OD450 | 0.0984 | 0.0502 | 0.1420 | 0.0383 |
| EU/mL | 0.0640025 | 0.05456119 | 0.07470771 | 0.052695692 |

**MATERIALS AND METHODS**

**S 1.1 Construction of recombinant plasmid and protein expression**

The correct plasmid (pET-NM) was transformed into *E. coli* BL21 (DE3) competent cells. For protein expression, the *E. coli* cells were grown at 37 °C in LB medium containing 100 μg/mL ampicillin until OD600 is around 0.8–1.0. Then 1 mM Isopropyl β-D-Thiogalactoside (IPTG , final concentration) was added to the culture for protein expression at 25°C for 12 h. For protein purification, the cells were harvested by centrifugation and lysed using High Pressure Homogenizer (PhD Technology LLC, USA). In order to obtain pure NM IBs, the insoluble pellets were resuspended in 30 ml of washing buffer (20 mM Tris, 300 mM NaCl, 1 mM EDTA, 1% Triton X-100, 1 M urea, pH 8.0) and extensively washed for three times. Finally, the inclusion bodies were washed with 20 mM Tris pH 8.0 to remove contaminating detergent and the purified inclusion bodies (NM-IBs) were used for subsequent solubilization study.

**S 1.2 Different solubilization methods**

Three methods of solubilization, traditional 8M urea-denature, freeze-thawing and one step-heating, were applied to obtain NM protein. In order to compare the solubilization efficiency of different methods, the same amount of purified NM inclusion body pellets was resuspended in Tris buffer at pH 8 containing different molar concentration of urea. For the traditional urea-denature method, the suspension containing 8 M urea was stirred for 30 minutes at room temperature and centrifuged (12,000 g, 20 minutes) to collect the supernatant. For the freeze-thawing method, the suspension containing 2 M urea was frozen at −20°C and thawed at room temperature, centrifuged at 12,000 g for 20 minutes to collect the supernatant. For the one step-heating method, the suspension containing 4 M urea was heated at 85°C for 20 min and collected the supernatant (12,000 g, 4°C, 20 minutes).

**S 1.3 Circular dichroism (CD) spectroscopy**

The CD spectra of the NM soluble proteins diluted in ultrapure water at concentrations from 0.08 to 1 mg/ml were acquired using a CD spectropolarimeter (Jasco, Japan) in a 1 mm path length. The spectra were recorded in the region of far-UV with a range of wavelength from 190 to 250 nm with a bandwidth of 0.5 nm at a scan rate of 50 nm/min. The CD spectral data were obtained in triplicate under the same conditions. The spectra were smoothed using Origin 9.0 software.

**S 1.4 Particles preparation and characterization**

To obtain aqueous solutions, lyophilized protein NM was dissolved in 6 M guanidiniumthiocyanate and subsequently dialyzed against a 10 mM Tris/HCl solution. The samples were centrifuged at 12,000 g to remove remaining aggregates and filtration (0.2 μm cellulose acetate syringe filter). Then, endotoxin was efficiently removed from protein samples by Endotoxin Removal Kit (Yeasen, China) for *in vitro/vivo* studies  and endotoxin content of the protein solution was determined using Chromogenic LAL Endotoxin Assay Kit (Beyotime, China) according to the instructions (Table S6, Supporting Information). The concentration of the resulting NM solution was determined by Pierce™ BCA Protein Assay Kits (Thermo Scientific, Rockford, USA).

For NM nanoparticles (NM-NPs) preparation，NM protein solution (2.5 mg/mL) was prepared (Supporting Information) and mixed with potassium phosphate (2 M, pH 8.0) in volumetric ratios of 1:10 using a pipette. Obtained spheres were centrifuged for 10 min at 12,000 g and then washed using ultrapure water for three times. The particles were re-dispersed in ultrapure water and particle concentrations (particles in mg/ml) were determined gravimetrically.

For NM nanoparticles (NM-NPs**)** characterization，the particle suspensions were sonicated for 5 min and diluted directly before the measurement to a concentration of 0.01 mg/ml in the corresponding buffer. The particle size, polydispersity index (PDI), and zeta-potential of NM particles were analyzed using a Nano Zetasizer (Malvern Instruments, Worcestershire, UK). The morphology and surface properties of nanoparticles were observed by scanning electron microscopy (HT7700, Hitachi, Japan). Briefly, one drop (10 µL) of the NPs was air-dried on a silicon slice and sputtered with gold. The nanoparticles were analyzed under a scanning electron microscopy with 15 kV of accelerating voltage. All measurements were performed three times in triplicate at 25°C.

**S 1.5 SDS-PAGE analysis the binding of lysozyme on NM-NPs.**

To further demonstrate the binding of lysozyme on NM-NPs, released lysozyme from loaded NM-NPs was analyzed by SDS-PAGE. The lysozyme loaded NM-NPs (Lyso-NM-NPs) was resuspended in 1 mL PBS and forced release was applied by boiling at 100°C for 10 min. After centrifugation, the sample (50 μL) from the supernatant was analyzed by SDS-PAGE to detect the released lysozyme. Also, samples (50 μL) taken from lysozyme solution before and after loading (supernatant after centrifugation) were analyzed by SDS-PAGE as control.

**S 1.6 Lysozyme activity test.**

Lysozyme can hydrolyze the β-1,4 glycosidic bond between the N-acetylmuramic acid of one monomer and the N-acetylglucosamine of the adjacent monomer, leading to cell wall instability and bacterial cell death. Thus, lysozyme can degrade bacterial solution, reduce turbidity and increase transparency, which can be measured the activity of lysozyme by luminosity changes. The lysozyme activity was measured according to Lysozyme (LZM) Assay kit, Turbidimetric method (Sangon Biotech, China) as instructions. Brifely, The prepared free lysozyme solution (2.5 ug/mL) and Lyso-NM-NPs (25ug/mL containing 2.5 ug/mL lysozyme) suspenssion 20 ul were added to individual wells to 200 ul of substrate solution in 96-well plates and the change of absorbance at 530 nm at 37 °C was measured by a microplated reader (Thermo Scientific, USA) for 20 min. Optical absorbance versus time data were compared to standard curves, which were plotted using linear regression of the change in absorbance over time versus unit active mass of lysozyme, to extrapolate active masses of lysozyme in the samples.

**S 1.7 The degradation behavior of NM-NPs**

The degradation behavior of NM-NPs was characterized by immersion tests in simulated gastric fluids (SGF) prepared according to 2015 Chinese pharmacopeia. The simulated gastric fluid (SGF) was obtained by dissolving 1% w/v pepsin in prepared dilute hydrochloric acid solution with pH adjusted to 2.5. Afterward, a static immersion test was carried out in a water bath incubator at 37 ^o^C for 24 h. Then, the morphology and surface properties of nanoparticles were observed by scanning electron microscopy (HT7700, Hitachi, Japan).

**S 1.8 Fluorescent labeling**

For the preparation of fluorescently labeled particles used for *in vivo/vitro* studies, the pre-prepared NM-NPs was suspended in 10mM Tris/HCl solution (2.5 mg/mL) to facilitate the coupling of Cy5.5 NHS ester (Aladdin, China). Then, a 10-fold molar excess of Cy5.5 (dissolved in DMSO) was added slowly to the particle suspension. After incubation for 24 h in the dark, the particles were centrifuged and washed with high pure water (HPW) for three times. Finally, the Cy5.5-labeled NM-NPs was suspended in 10 mM phosphate buffer (PBS) at pH 7.4 with a concentration of 1 mg/1mL. In order to detect whether Cy5.5 binds to the nanoparticles, we measured the fluorescence before and after the binding of nanoparticles with an emission at 675nm and excitation at 693nm by a microplated reader (Thermo Scientific, USA). Also, the fluorescence of the the washing buffer at the third time was detected. The following results showed the successful labeling of Cy5.5 on nanoparticles (Table S4 and Figure S1).

**S 1.9 Different administration routes**

For oral administration, mice fasted overnight with free access to water before particle administration with a disposable animal-feeding needle. All mice fasted and had free access to water throughout the experiment. By s.c. injection, mice were injected with 100 μl PBS containing different formulations into the dorsal flanks. For i.m. injection, mice were injected with 100 μl PBS containing different formulations into the right hind leg. For i.v. injection, mice were injected with 100 μl PBS containing different formulations into the tail vein.

**S 1.10 Bone marrow derived cells**

Briefly, the femurs and tibias were rinsed in 70% ethanol. Red blood cell (RBC)-depleted Balb/c bone marrow cells were seeded into 24-well plates with a density of 1× 10^5^ cells per well in complete RPMI media supplemented with 20 ng/mL murine recombinant **granulocyte-macrophage colony stimulating factor** (GM-CSF) and 20 ng/mL murine recombinant Interleukin-4 (IL-4). At day 3 and 6, half of the culture supernatant was replaced with fresh RPMI 1640 medium containing same GM-CSF and IL-4. Loosely and nonadherent cells were collected and used as inactivated BMDCs on day 8.

**S 1.11 Determination of lysozyme-specific IgG and IgG subclasses**

Serum from immunized mice was assayed by ELISA to determine levels of antigen-specific antibodies (total IgG, IgG1, and IgG2a). Briefly, 96-well microtiter plates were coated with 100 μL of 10 μg/mL lysozyme solution overnight at 4°C and then blocked with 3% BSA (Sigma, USA) in PBS. Then plates were washed with PBS containing 0.1% Tween-20 (PBST) and incubated with 100 μL of appropriate sera dilution (diluted 1:40 in PBST containing 0.1% [m/v] BSA) for 1 h at 37°C. After washing, the plates were incubated with 100 μL horseradish peroxidase-conjugated anti-mouse IgG, IgG1 and IgG2a antibodies (Sigma, USA, diluted 1:10,000 in assays of IgG, IgG1 and IgG2a) for 1 h at 37°C. Thereafter, the plates were washed with PBST and added 100 μL of 3,3’,5,5’-tetrame-thylbenzidine (TMB) substrate to each well for 20 min at room temperature. The reaction was stopped by adding 50 μL of 2 M H_2_SO_4_ and optical density (OD 450 nm) was measured by a microplated reader (Thermo Scientific, USA). All serum samples were tested in duplicate.

**S 1.12 Determination of Cytokine Levels**

Spleens were harvested from vaccinated mice of each group (n=5 animals per group) 7 days after the third immunization and homogenized aseptically in sterile PBS. Spleens were homogenized aseptically in sterile PBS and treated with ACK buffer for 5 min to lyse the red blood cells. The splenic cells were then suspended in complete DMEM (containing 5% FCS, 0.1% L-glutamine, 1% HEPES, 0.1% 2ME, and 0.1% gentamicin). For determination of cytokine production, splenocytes were collected and cultured in 24-well microliter plates with 3 × 10^5^ cells/well in triplicates in the presence or absence of 10 μg/mL lysozyme and incubated for 72 h at 37°C in 5% CO_2_. Finally, culture supernatant samples were collected, and cytokine concentration (Interferon gamma, IFN-γ and Interleukin-4, IL-4) was measured by the ELISA kit (eBioscience, USA) according to the manufacturer’s instructions. Briefly, 96-well microplates were covered with mouse IL-4 and IFN-γ capture antibodies separately overnight at RT. Following washing and blocking, the collected supernatant was added  and incubated for 2 h at 37 °C. Then, mouse IL-4 and IFN-γ detection antibodies were added and incubated for 2 h at 37 °C. Finally, the peroxidase substrate system was added for incubation 30 min at 37 °C and 1% SDS was used for stopping the reaction. The absorbance of 96-well microplates was read at 405 nm and the concentration was calculated with a standard curve. All the measurements were run in duplicates.

**S 1.13 Flow cytometry**

For the induction of dendritic cell maturation, the mice were sacrificed at 48 h after subcutaneous administration. Draining lymph nodes were isolated, ground up and filtered through a 70-μm cell screen mesh to obtain single cell suspension. Cells were collected by centrifugation, washed with PBS, then stained for 30 min at 4 °C with a mixture of anti-mouse antibodies (PE anti-CD11c, PerCP Cy5.5 anti-CD80 and FITCanti-CD86; all from eBioscience). Stained cells were washed twice with PBS, and expression of CD80 and CD86 on CD11c^+^ DCs was determined by flow cytometry (Beckman Coulter, USA).

**S 1.14 Cell cytotoxicity assay**

Toxicity of the unloaded NM-NPs for BMDCs was evaluated using the CCK-8 Cell Proliferation Assay Kit (NCM biotech, China). Cells were plated at 5×10^4^ cells/well in 96-well plates and cultured in a humidified atmosphere containing 5% CO_2_ at 37 °C for 24 h. Then 10 μL of NM-NPs were added to the 96-well plates in triplicate to yield final concentrations of 50, 100, 250, 300, 400 and 500 μg mL−1, PBS as a control. After 24 h incubation, 10 μL CCK-8 solution was added to each well, and the plates were incubated for an additional 4 h at 37 °C. The viability of cells was measured by detecting absorbance at 450 nm using a microplate reader (Thermo Scientific, USA). The experiments were repeated three times.

Cell viability was calculated according to the equation,

Cell viability (%) = (OD_sample_/OD_control_) × 100%

where OD_control_ was obtained in the absence of added nanoparticls. Untreated cells served as 100% cell viability.

**S 1.15 Unspecific immune responses assay**

In order to investigate whether NM-NPs lead to unspecific dendritic cell maturation in themselves. BMDCs were seeded on 12-well plates at a density of 1×10^6^ cells per well. After 24 h incubation with free lysozyme (final lysozyme concentration was 20 ug mL^−1^), empty NM-NPs (final NM-NPs concentration was 200 ug mL^−1^), NM-NPs containing lysozyme (final NM-NPs concentration was 200 ug mL^−1^ containing lysozyme 20 ug mL^−1^) and PBS, respectively, the BMDCs were collected. The BMDCs were then stained with fluorescence-labeled monoclonal antibodies for 30 min at room temperature and washed twice with PBS. The expression of CD80 and CD86 on CD11c^+^ DCs was determined by flow cytometry (Beckman Coulter, USA).

**S 1.16 NM-NPs tolerance in mice**

The mice were grouped with three mice per group and repeatedly treated with 250 μg NM-NPs suspended in 100 μl PBS for 3 days by intravenous injection. The control group was equally treated with 100 μL PBS. Then, the mice were euthanized via decapitation and the heart, kidney, liver, lung, spleen and small intestines were collected from each animal after 24 h of the last treatment. Then, the tissues were fixed in 10% phosphate-buffered formalin, paraffin embedded, sectioned and stained with H&E for histological examination.
